# Mitochondrial targeting of *Candida albicans* SPFH proteins and requirement of stomatins for SDS-induced stress tolerance

**DOI:** 10.1128/spectrum.01733-24

**Published:** 2024-12-06

**Authors:** Hyunjeong Kim, Marienela Y. Heredia, Xiao Chen, Maisha Ahmed, Mohammad Qasim, Tracy L. Callender, Aaron D. Hernday, Jason M. Rauceo

**Affiliations:** 1 Department of Sciences, John Jay College of the City University of New York, New York, New York, USA; 2 Department of Molecular and Cellular Biology, School of Natural Sciences, University of California, Merced, California, USA; 3 Department of Biology, Farmingdale State College of the State University of New York, Farmingdale, New York, USA; University at Buffalo, State University of New York, Buffalo, New York, USA

**Keywords:** *Candida albicans*, prohibitin, mitochondria, surfactant, stomatin

## Abstract

The SPFH (stomatin, prohibitin, flotillin, and HflK/HflC) protein superfamily is conserved across all domains of life. Fungal SPFH proteins are required for respiration, stress adaptation, and membrane scaffolding. In the yeast *Candida albicans*, stomatin-like protein 3 (Slp3) forms punctate foci at the plasma membrane, and *SLP3* overexpression causes cell death following exposure to the surfactant, SDS, and the oxidative stressor, H_2_O_2_. Here, we sought to determine the cellular localization and functionally characterize stomatin-like protein 2 (Slp2), prohibitin-1 (Phb1), prohibitin-2 (Phb2), and prohibitin-12 (Phb12) in *C. albicans*. Cytological and western blotting results showed that Slp2-Gfp/Rfp and prohibitin-Gfp fusion proteins localize to the mitochondrion in yeast cells. Growth assay results did not identify any respiration defects in a panel of stomatin and prohibitin mutant strains, suggesting that SPFH respiratory function has diverged in *C. albicans* from other model eukaryotes. However, a *slp2Δ*/*Δ*/*slp3Δ*/*Δ* double mutant strain grew poorly in the presence of 0.08% SDS, accumulated intracellular reactive oxidative species, and displayed aberrant ergosterol distribution in the plasma membrane. These phenotypes were not observed in *slp2Δ*/*Δ* or *slp3Δ*/*Δ* single mutants, indicating a possible indirect genetic interaction between *SLP2* and *SLP3*. In addition, *slp2Δ*/*Δ* and *slp2Δ*/*Δ*/*slp3Δ*/*Δ* mutant strains were slightly resistant to the antifungal drug, fluconazole. Collectively, these findings reveal the cellular localization of Slp2, Phb1, Phb2, and Phb12, highlight the significance of stomatins in *C. albicans* SDS stress tolerance, and, for the first time, associate stomatins with antifungal resistance.

**IMPORTANCE:**

Stomatins and prohibitins coordinate respiration and stress adaptation in fungi. Invasive mycoses caused by *Candida albicans* are a significant cause of morbidity, and candidemia patients show high mortality rates worldwide. Mitochondria are essential for *C. albicans* commensalism and virulence, and mitochondrial proteins are targets for antifungal interventions. *C. albicans* encodes five SPFH proteins: two stomatin-like proteins and three prohibitins. We have previously shown that Slp3 is important for *C. albicans* adaptation to various types of environmental stress. Moreover, synthetic compounds that bind to mammalian prohibitins inhibit *C. albicans* filamentation and are fungicidal. However, there is limited information available regarding the remaining SPFH proteins. Our findings show that mitochondrial localization of SPFH proteins is conserved in *C. albicans*. In addition, we demonstrate the importance of stomatins in plasma membrane and mitochondrial stress tolerance.

## INTRODUCTION

*Candida albicans* colonizes a variety of human mucosal surfaces as a commensal fungus. Under permissive conditions, *C. albicans* can become pathogenic, causing superficial oral and vaginal infections and forming multidrug-resistant biofilms on indwelling medical devices. In severe infection cases, disseminated candidiasis is often fatal in immunosuppressed patients (1). This unique lifestyle of *C. albicans*, coupled with a lack of an efficient sexual reproductive cycle, requires robust adaptive mechanisms to survive diverse environmental niches. Thus, *C. albicans* has evolved novel organellar functions beyond those conserved across all eukaryotes to thrive as both a commensal and a pathogen. The role of the mitochondria in *C. albicans* virulence exemplifies this strategy (2–4).

In fungi, the mitochondrion governs respiration as well as non-respiratory processes, including lipid synthesis and reactive oxidative species (ROS) production (5). *C. albicans* mitochondria are also associated with virulence determinants, including biofilm development, drug susceptibility, filamentation, and cell wall biogenesis (2–4, 6, 7). Moreover, the fungal-specific mitochondrial complex I (CI) regulator, Goa1, as well as CI subunits, Nuo1, and Nuo2, are essential for respiration and virulence (8, 9). These attributes emphasize the clinical potential of targeting mitochondrial proteins for novel fungal interventions and the importance of understanding the molecular mechanisms underlying mitochondrial function. Indeed, the widely used antifungal drug, fluconazole, localizes to the mitochondria in yeast cells and inhibits synthesis of the critical plasma membrane component, ergosterol (7).

SPFH (stomatin, prohibitin, flotillin, and HflK/HflC) integral membrane proteins are widely conserved and function mainly as scaffolds (10–12). SPFH family members are pleiotropic, coordinating diverse processes including mechanosensation, transport, signaling, and respiration (13–20). Attenuation of human prohibitins is associated with Alzheimer’s disease and various types of cancer (21). Molecular details underlying SPFH protein function have been derived in part from studies investigating mitochondrial SPFH proteins in several species. Human stomatin-like-protein 2 (SLP-2) and prohibitin protein 2 (PHB2) localize to the mitochondrial inner membrane to support respiratory chain complex formation and mitophagy (19, 22, 23). Pathogenesis of the rodent malaria-causing parasite, *Plasmodium berghei*, is abrogated when the mitochondrial membrane prohibitin-like protein, PHBL, is disrupted (24). In the model yeasts *Saccharomyces cerevisiae* and *Schizosaccharomyces pombe*, the prohibitins, Phb1 and Phb2, are required for mitochondrial respiratory and non-respiratory functions such as lipid synthesis, autophagy, and drug resistance (15, 18). Furthermore, in *S. cerevisiae*, Phb1 and Phb2 exist as ring structures bound to the mitochondrial inner membrane, where they interact with Mdm33 to influence mitochondrial morphology. Lastly, Phb1 and Phb2 chaperone the mitochondrial COX complex and interact with Atp10 and Atp23 to form the F_1_F_o_ ATP synthase complex (18, 25–27). These observations highlight the importance of SPFH proteins in mitochondrial function across several kingdoms of life.

We previously discovered a role for a stomatin protein in *C. albicans*. Gene expression and cellular analyses revealed that *SLP3* (stomatin-like-protein 3) transcription was significantly elevated in cells treated with oxidative, cell wall, or plasma membrane stress agents (28, 29). Slp3 formed visible punctate foci along the plasma membrane and localized within the vacuolar lumen in yeast-phase cells specifically, as it was not expressed in hyphal cells (28). A homozygous *slp3Δ*/*Δ* null mutant strain did not have an apparent phenotype when examined in a variety of cellular and biochemical tests. However, *SLP3* overexpression triggered apoptotic-like death specifically following prolonged exposure to SDS, H_2_O_2_, or hyphae-inducing conditions (28). *C. albicans* mitochondrial function is heavily implicated in the yeast-to-hyphae transition and oxidative stress adaptation (9, 30, 31). Recently, pharmacologically active triazine-based compounds that bind to human prohibitins were demonstrated to be fungicidal in *C. albicans* and non-*albicans Candida* species, thus indicating a need to further understand SPFH function in pathogenic yeast (32).

While these findings identified a role for stomatins in the *C. albicans* oxidative stress response and yeast-to-hyphae transition, the molecular function of Slp3 remains elusive. Also, the localization and function of the remaining four SPFH family members (*SLP2*, *PHB1*, *PHB2*, and *PHB12*) are unknown. Here, we utilized a cellular and molecular approach to determine the location of Slp2, Phb1, Phb2, and Phb12 in *C. albicans*. Furthermore, our genetic phenotyping results show that both *SLP2* and *SLP3* are required for the adaptive response to SDS-induced cytotoxicity. Lastly, we identify a role for stomatins in antifungal drug resistance.

## MATERIALS AND METHODS

### Yeast strains and media

*C. albicans* wild-type reference strains BWP17 and DAY286 were used to construct strains containing SPFH fluorescent fusion proteins. The wild-type reference strain SN250 was used to construct *C. albicans* mutant strains. All strains used in experimental assays were isogenic, and genotypes are listed in the Appendix S1. Yeast starter cultures were prepared in YPD^+uri^ (1% yeast extract, 2% peptone, 2% dextrose, and 80 mg/L uridine) or YPD media at 30°C with shaking at 225 rpm unless otherwise noted.

### Yeast strain construction

Oligonucleotide sequences are listed in the Appendix S1. Yeast transformations followed the lithium acetate protocol and were verified by colony PCR (33). SPFH fluorescent protein-tagged strains were constructed by PCR-mediated homologous recombination (34). We used plasmid pMG1646 (35) as a template to produce an amplicon containing *GFP*-, *HIS1*-, and SPFH gene-specific sequences. SPFH-*GFP* amplicons were transformed independently into *C. albicans* strain DAY286 and integrated directly following the final amino acid encoding codon to produce strains MYH05 (*PHB1-GFP*), MYH11 (*SLP2-GFP*), MYH20 (*PHB2-GFP*), and MA01 (*PHB12-GFP*). We used plasmid pMG2169 (36) to produce an *SLP2-RFP* amplicon and transform it into strain BWP17 to produce strain MYH45.

Overexpressing yeast strains were constructed as previously described (37). Briefly, to construct the *P_TDH3_SLP2-GFP* and *P_TDH3_SLP2-RFP* overexpressing strains, MYH31 and MYH53, respectively, we used plasmid pCJN542 as a template to produce an amplicon containing the glyceraldehyde-3-phosphate dehydrogenase (*TDH3*) promoter sequences, nourseothricin resistance gene (*NAT*), and *SLP2*-specific sequences. The amplicon was transformed into the *C. albicans SLP2-GFP* strain (MYH11) and *SLP2-RFP* strain (MYH53) and integrated directly upstream of the *SLP2* start codon. To construct the *P_TDH3_PHB1-GFP*, *P_TDH3_PHB2-GFP*, and *P_TDH3_PHB12-GFP* overexpressing strains, SF05, SF20, and MA06, PCR amplification and yeast strains MYH05, MYH20, and MA01 were transformed as described above.

CRISPR/Cas9 genome editing was performed as previously described (32) to create SPFH single- and double-mutant strains. Add-back (complemented) strains were generated by targeting Cas9 to cut the AddTag sequences and reintroducing the native ORF at both alleles of the native locus via homologous recombination with a donor DNA fragment containing the previously deleted ORF along with approximately 250 bp of flanking homology. Add-back genotypes were confirmed by colony PCR as previously described (38).

### Microscopy and flow cytometry

Approximately 2.5–5.0 µL of cell suspension was spotted onto poly-lysine coated slides for microscopic analysis. Samples were observed at a total magnification of 1,000× with an EVOS M5000 imaging system equipped with the appropriate light cubes for fluorescent visualization and on-board software for image review and analysis (ThermoFisher Scientific). Experiments were repeated at least three times unless otherwise noted, and data presented are representative of one experiment.

Flow cytometry experiments were performed using an Attune NxT Flow Cytometer (Life Technologies) equipped with a 50 mW, 488 nm LED laser, and 530/30 nm emissions filters to quantitatively measure yeast cell fluorescence. Data were analyzed using Attune NxT Software v2.2. We gated out all events with fluorescence below the maximum value for wild-type untagged control cells to account for potential yeast cell auto-fluorescence, unless otherwise noted. For each assay, three technical replicates were analyzed. Experiments were repeated at least three times unless otherwise noted, and data presented are representative of one experiment. Ten thousand events were collected for each sample, and the mean fluorescent intensity (MFI) was recorded. The average MFI was determined, and statistical differences between samples were analyzed in paired *t*-tests.

### Protein localization assays

Designated *C. albicans* strains were streaked from frozen stocks onto fresh YPD or YPD^+uri^ plates. Single colonies were selected, inoculated into 5.0 mL YPD or YPD^+uri^, and grown at 30°C with shaking at approximately 225 rpm for 16–20 hours. Yeast cells were diluted to a final OD_600nm_ of approximately 0.20 in 5.0 mL of fresh YPD or YPD^+uri^. Cells were incubated to an OD_600nm_ of approximately 0.80–1.00 (mid-late exponential phase), and fluorescence was examined microscopically. Samples were collected following 16–20 hours of incubation to examine stationary-phase yeast cells. To quantify cellular fluorescence, yeast cells were harvested at the designated time, washed with 1× phosphate-buffered saline (PBS) solution pH 7.0, resuspended in 1.0 mL of 1× PBS, and analyzed in the flow cytometer.

For mitochondrial co-localization tests, cell cultures were prepared as described above to reach the designated phase of the yeast life cycle. Nine hundred ninety microliter of yeast cell suspension was mixed with 10 µL of MitoTracker Red or MitoTracker Green (ThermoFisher Scientific) to a final concentration of 100 nM. Samples were incubated at 37°C for 45 minutes to 1 hour in the dark, washed twice with dH_2_O, resuspended in approximately 100–200 µL of dH_2_O, and examined under the microscope.

To visualize the nucleus, 500 µL of exponential- or stationary-phase samples was collected and mixed with 50 µL of 37% formaldehyde. After fixation, the cells were washed three times with 1× PBS and resuspended in approximately 100 µL of 1× PBS. Two drops of NucBlue Fixed Cell Ready solution (ThermoFisher Scientific) were added to the yeast cell suspension. Samples were incubated for 5 minutes at room temperature and immediately viewed. To visualize the cell wall, plasma membrane, or vacuole/endosome, cells were stained with 100 µg/mL Calcofluor White, 200 µg/mL Filipin, or 160 µM FM 4–64, respectively, as previously described (28, 39). To visualize lipid droplets, cells were washed with 1× PBS and incubated with the HCS LipidTOX Green Neutral Stain according to the manufacturer’s instructions (Invitrogen).

Yeast cells from overnight cultures were harvested and diluted to an OD_600nm_ of 0.20 in 5.0 mL of Spider medium (1% nutrient broth, 1% mannitol, 11.5 M potassium phosphate, and pH 7.2) to examine protein localization in the yeast-to-hyphae transition. The cultures were grown for approximately 3–6 hours at 37°C and microscopically examined. Mitochondrial co-localization tests with hyphal cells were performed as described above.

### Growth assays

Growth assays on solid nutrient growth medium were performed as previously described (40). Briefly, *C. albicans* overnight cultures were diluted to an OD_600nm_ of 3.00. Cells were serially diluted, spotted onto YPD nutrient plates, incubated at 30°C, and photographed after 1–3 days of growth. YPD plates supplemented with the designated compound were used to monitor growth under environmental stress.

Yeast growth kinetics were monitored as previously described (37). Briefly, cells from overnight cultures were diluted to an OD_600nm_ of 0.20 in 200 µL YPD in a 96-well plate. To determine the effect of environmental stress on yeast growth, cells were incubated with YPD supplemented with the designated compounds. Samples were grown for 24 hours with shaking at 30°C, and the OD_600nm_ was acquired every 30 minutes using a Synergy Mx plate reader (Biotek). Minimum inhibitory concentration (MIC) tests were performed as previously described (32).

### Mitochondrial assays

To examine growth under non-fermentable conditions, yeast cells were prepared as described in the growth assay section and spotted onto YPD or complete synthetic medium nutrient plates containing 2%–8% glycerol or 2% lactate as carbon sources. Plates were incubated at 30°C and photographed after 2–4 days of growth. To examine the effect of respiratory inhibitors on growth, yeast cells were spotted onto YPD nutrient plates supplemented with Antimycin A at the designated concentration, 0.5 mM salicylhydroxamic acid (SHAM), 1.0 mM sodium nitroprusside (SNP), or 0.5 mM SHAM + 1.0 mM SNP. Yeast growth was also monitored under identical conditions in microplate liquid assays as described in the growth assays section.

ROS intracellular levels were examined in early stationary-phase yeast cells as previously described (28). Briefly, samples were cultured as described in the protein localization section for approximately 3 and 20 hours and diluted to an OD_600nm_ of 0.20 in 5.0 mL of fresh YPD supplemented with 0.08% SDS (or dH_2_O as a control). Cells were incubated at 30°C with shaking for 3 or 20 hours. The samples were harvested and washed with 1× PBS, and approximately 1.0–5.0 × 10^6^ cells were resuspended in 1.0 mL of 1× PBS. Next, the cells were incubated with 5 µg/mL dihydrorhodamine 123 (DHR-123; Sigma) or dH_2_O as a control for 10 minutes in the dark at room temperature and then analyzed by flow cytometry.

### Slp2p primary sequence analysis

Post-translational modification and organelle targeting predictions were made using InterProScan (41), MitoFates (42), and TPpred 2.0 (43) provided by the bioinformatics resource portal ExPASy (https://www.expasy.org/). Protein schematics were constructed using Illustrator for Biological Sequences, version 1.0 (44).

### Mitochondrial isolation

Crude mitochondria were isolated using the Yeast Mitochondrial Isolation kit (Abcam) following the manufacturer’s instructions. Yeast cultures were grown overnight in 2.0 mL of YPD to stationary phase. The following day, cultures were diluted in 25–50 mL of YPD and grown to mid-late exponential phase (OD_600_ of 0.80–1.00). Next, 10 mL of each culture was harvested at 3,000 rpm for 5 minutes, and the cell pellets were washed in 20 mL ultrapure water. Cell pellets were resuspended in company-supplied Buffer A supplemented with a final concentration of 10 mM DTT and incubated for 10 minutes at 30°C with gentle shaking. Cells were centrifuged at 1,500× g for 5 minutes. Yeast cells were resuspended in company-supplied Buffer B with lysis enzymes and incubated with shaking for 15 minutes at 30°C. Cells were centrifuged at 1,500× g for 5 minutes, and cell pellets were resuspended in 1.0 mL of homogenization buffer with protease inhibitors and homogenized 15 times while on ice. The cell extract was centrifuged at 600× g for 5 minutes at 4°C. The supernatant was collected and centrifuged at 600× g for an additional 5 minutes at 4°C. The final supernatant was centrifuged at 12,000× g for 10 minutes at 4°C. The remaining pellet containing extracted mitochondria was resuspended in company-supplied storage buffer.

### Western blots

Protein sample concentrations were determined in BCA assays according to the manufacturer’s instructions. Samples were mixed with 2× SDS protein sample buffer and heated at 95°C for 5 minutes. Approximately 20 µg of each sample was fractionated on 12% TGX FastCast acrylamide SDS gels (BioRad) at 150 V for 65 minutes. Proteins were transferred to polyvinylidene fluoride (PVDF) membranes at 100 V for 40 minutes. After transfer, PVDF membranes were blocked with 5.0% nonfat milk in TBST (5.0 M NaCl, Tween 20, 1.0 M Tris-HCl, and pH 7.5) for 30 minutes at room temperature. Membranes were then incubated with anti-GFP-horseradish peroxidase (HRP) conjugated monoclonal primary antibodies (Invitrogen) diluted in 5.0% milk/TBST at a 1:5,000 dilution for 16–20 hours at 4°C.

Actin was detected from crude mitochondrial extracts of each yeast strain to serve as a loading control. Anti-actin antibodies (Invitrogen) were prepared at a 1:2,500 dilution in 5.0% nonfat milk/TBST and incubated overnight with blocked PVDF membranes at 4°C with gentle shaking. Next, the anti-actin solution was removed, and the membrane was rinsed with TBST. The membrane was incubated with goat anti-mouse IgG-HRP (Cell Signaling) antibodies used at a 1:1,000 dilution in milk/TBST for 1 hour at room temperature with shaking. All blots were developed using Clarity Western ECL reagent following the manufacturer’s instructions (BioRad).

## RESULTS AND DISCUSSION

### Localization of *C. albicans* prohibitins

**Fig 1:**
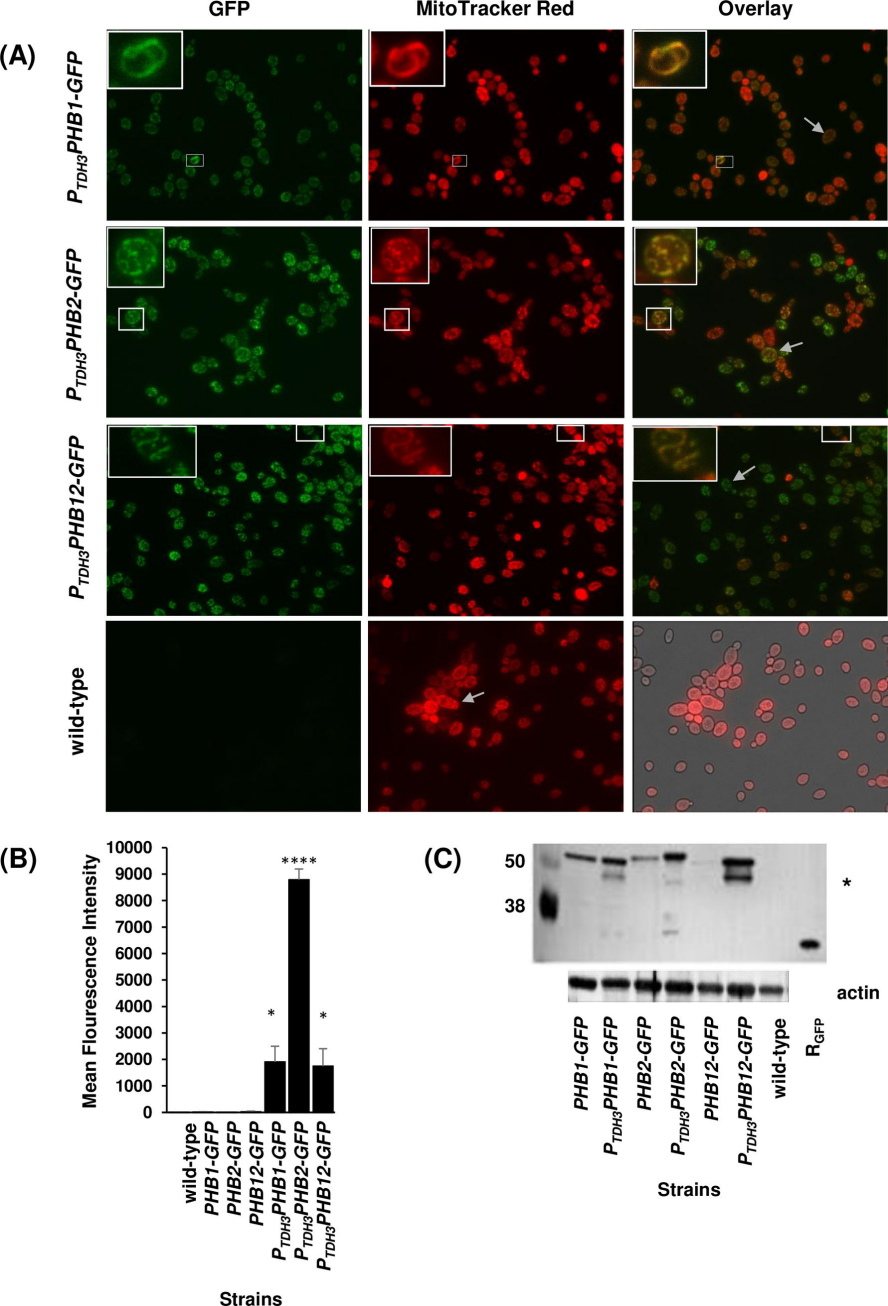
*C. albicans* prohibitin localization. (**A**) Samples of exponential-phase *P_TDH3_PHB1-GFP*, *P_TDH3_PHB2-GFP*, and *P_TDH3_PHB12-GFP* overexpressing cells and untagged wild-type control cells were treated with 100 nM MitoTracker Red. Cells were viewed under bright-field and fluorescent microscopy. The right panels show an overlay of GFP and MitoTracker Red fluorescence. The white boxes depict cells shown in the inset images. White arrows in the overlay images are used to depict intracellular mitochondrial networks. (**B**) Cellular fluorescence of prohibitin-GFP and prohibitin-GFP overexpressing cells was quantified with flow cytometry. For each assay, three technical replicates were analyzed. Untagged wild-type control cells were analyzed at identical time points and served as a negative fluorescent control. Experiments were repeated at least three times, and data presented represent one representative experiment. *P < 0.05 and ****P < 0.001 when compared to the isogenic parental strain. (**C**) Crude mitochondrial extracts were prepared from the designated strains, and Phb1-Gfp, Phb2-Gfp, and Phb12-Gfp were identified on western blots. Purified recombinant GFP protein (R_GFP_) was included as a positive control for anti-GFP antibody/antigen binding, and mitochondrial extracts were prepared from the untagged wild-type reference strain to serve as a negative control for antibody binding. The asterisk denotes potentially degraded prohibitin-Gfp protein fragments. Bottom immunoblot: actin was identified from mitochondrial extracts to serve as a loading control.

*S. cerevisiae* Phb1 and Phb2 localize to the mitochondrion (25). Therefore, we hypothesized that *C. albicans* prohibitins would also localize to the mitochondrion. *C. albicans* strains encoding GFP-tagged Phb1, Phb2, and Phb12 were visualized using fluorescent light microscopy. Phb1-Gfp, Phb2-Gfp, and Phb12-Gfp intracellular fluorescence were slight to undetectable in exponential- and stationary-phase yeast cells and throughout the yeast-to-hyphae transition. These findings were not due to yeast cell auto-fluorescence because cellular fluorescence was not observed in untagged wild-type control cells (Fig. 1A). The absence of fluorescence in *PHB1-GFP*, *PHB2-GFP*, and *PHB12-GFP* yeast cells contrasted with previous findings with *C. albicans* Slp3. In those studies, Slp3-Yfp fluorescence was apparent throughout the yeast life cycle, followed by treatment with various chemical stress agents (28).

We reasoned that the undetectable Phb1-Gfp, Phb2-Gfp, and Phb12-Gfp fluorescence was due to low gene expression in cells cultured in nutrient-rich media. Thus, we examined the transcriptional profile of *PHB1*, *PHB2*, and *PHB12* from several independent genome expression data sets (45). *PHB1*, *PHB2*, and *PHB12* expression remained at basal levels under growth conditions comparing exponential- and stationary-phase cells grown in nutrient media (46) or when cells were challenged with mitochondrial (SDS and H_2_O_2_) (47, 48), cell wall (caspofungin) (40, 49), osmotic (NaCl) (29), or plasma membrane (fluconazole) (50) stress. In addition, cellular fluorescence was not observed in aging cell cultures (7–10 days incubation) containing fermentable or non-fermentable carbon sources (our unpublished data).

Since basal prohibitin levels were insufficient to visualize by light microscopy, we overexpressed *PHB1-GFP*, *PHB2-GFP*, and *PHB12-GFP*. Previous studies with *C. albicans* Slp3 demonstrated that overexpression of *SLP3-YFP* did not alter protein localization, supporting this approach (28). Microscopy images and flow cytometry results show that cellular fluorescence was significantly greater in *P_TDH3_PHB1-GFP*, *P_TDH3_PHB2-GFP*, and *P_TDH3_PHB12-GFP* overexpressing exponential- and stationary-phase cells compared to parental strains and the untagged wild-type control strain (Fig. 1A and B; Fig. S1). Phb1-Gfp, Phb2-Gfp, and Phb12-Gfp were visible at the cell periphery (mostly in exponential- phase cells) and within the cell (mostly in stationary-phase cells; Fig. 1A; Fig. S1A).

To determine whether *C. albicans* prohibitins localize to the mitochondria, *P_TDH3_PHB1-GFP*, *P_TDH3_PHB2-GFP*, and *P_TDH3_PHB12-GFP* exponential- and stationary-phase cells were stained with the mitochondria-labeling dye, MitoTracker Red, and examined via fluorescence microscopy. MitoTracker Red fluorescent images show intracellular tubular morphology and cell peripheral labeling that is characteristic of yeast mitochondria (Fig. 1A; Fig. S1A). MitoTracker Red/GFP overlay images show that Phb1-Gfp, Phb2-Gfp, and Phb12-Gfp co-localize at the mitochondria, indicated by the yellow/orange color (Fig. 1A; Fig. S1A).

To confirm prohibitin mitochondrial localization, mitochondria extracts were prepared from prohibitin-Gfp strains and the untagged parental reference strain. Phb1-Gfp (58 kDa), Phb2-Gfp (61 kDa), and Phb12-Gfp (58 kDa) were detected on immunoblots probed with anti-Gfp-HRP antibodies at the expected molecular masses (Fig. 1C). Furthermore, these western blotting results were in concordance with microscopy and flow cytometry findings, whereby greater prohibitin protein levels were observed in overexpressing prohibitin strains compared to parental strains (Fig. 1A and B). We also observed several lower molecular weight bands in samples collected from prohibitin overexpressing strains. The presence of these bands was not a consequence of non-specific binding of the anti-GFP antibodies to an unknown protein because these bands were absent in the untagged wild-type reference strain (Fig. 1C). It is possible that these bands represent fusion protein degradation products.

Prohibitin mitochondrial targeting was observed in the germ tube of *P_TDH3_PHB2-GFP* cells undergoing the yeast-to-hyphae transition (Fig. S1B). However, mitochondrial targeting was undetermined in *P_TDH3_PHB1-GFP* and *P_TDH3_PHB12-GFP* hyphal cells. Notably, aberrant filament structure and apoptotic-like cell death were not observed in *P_TDH3_PHB2-GFP* overexpressing hyphal cells, as previously reported for overexpressing *P_TDH3_SLP3*-YFP cells (28), suggesting that prohibitins are not yeast-phase-specific genes compared to *SLP3*. Additionally, transcription profiling results from a different study showed that prohibitin transcription is not downregulated during the yeast-to-hyphae transition (51). Collectively, our cytological and western blot results demonstrate that prohibitin mitochondrial localization is conserved in *C. albicans* and suggest that the expression of prohibitin genes is tightly regulated.

### Localization of *C. albicans* Slp2

**Fig 2:**
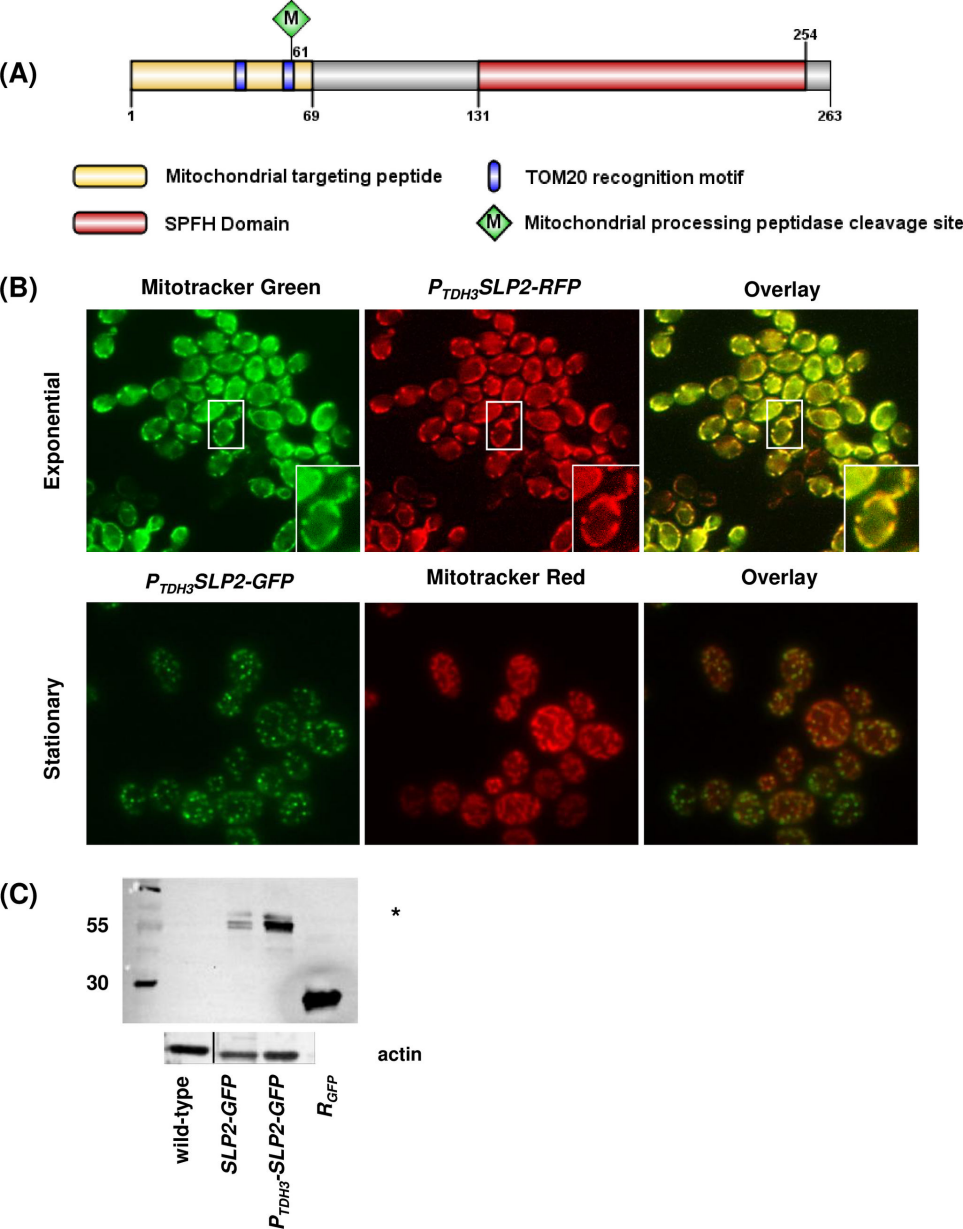
*C. albicans* Slp2 localization. (**A**) Schematic representation of Slp2p amino acid sequence. Image is drawn to scale. The predicted N-terminal mitochondrion localization region, SPFH domain, TOM20 recognition sites, and mitochondrial processing peptidase cleavage site are featured. (**B**) Exponential-phase (top row) and stationary-phase cells (bottom row) of the *P_TDH3_SLP2-GFP* and *P_TDH3_SLP2-RFP* overexpressing strains were treated with 100 nM MitoTracker Green (for *P_TDH3_SLP2-RFP* cells) or 100 nM MitoTracker Red (for *P_TDH3_SLP2-GFP* cells). Cells were viewed under fluorescent (RFP and GFP) microscopy. The right panels show an overlay of the fluorescent tag and MitoTracker dye. The white box seen in *P_TDH3_SLP2-RFP* images highlights the cell depicted in the inset image. (**C**) Mitochondrial extracts were prepared from the designated strains, and Slp2-Gfp was identified on western blots. Purified recombinant GFP protein (R_GFP_) was included as a positive control for anti-GFP antibody/antigen binding, and mitochondrial extracts were prepared from the untagged wild-type reference strain to serve as a negative control for antibody binding. The asterisk denotes potentially degraded Slp2-Gfp protein fragments. Bottom immunoblot: actin was identified from mitochondrial extracts to serve as a loading control. The actin loading control blot shows a composite image of the same blot, where the wild-type sample was moved from lanes 3 to 1 for alignment with the GFP blot.

*C. albicans SLP2* shares significant homology with human SLP-2 which is found in the mitochondrion (28). Bioinformatic analyses of the *C. albicans* Slp2 amino acid primary sequence using the programs InterProScan, MitoFates, and TPpred 2.0 were performed to identify putative mitochondrial targeting sequences. We identified a mitochondrial targeting sequence in the N-terminal region of Slp2. In addition, we identified two TOM20 recognition motifs that may facilitate protein import from the outer mitochondrial membrane and a mitochondrial processing peptidase site (Fig. 2A).

The presence of putative mitochondrial targeting sequences predicts that *C. albicans* Slp2 localizes in the mitochondria. Notably, yeast cells expressing *SLP2-GFP* displayed low levels of intracellular fluorescence (Fig. S2A). *SLP2* transcription was not increased when cells were grown in the presence of various additives (29, 40, 45–47, 49, 50), implying that *SLP2* gene expression, like the prohibitin-encoding genes, may also be tightly regulated. Therefore, a *P_TDH3_SLP2-GFP* overexpressing strain was constructed to determine protein localization.

Slp2-Gfp expression increased approximately five-fold in the overexpressing strain (Fig. S2B). Co-localization microscopic analysis with MitoTracker Red showed that Slp2-Gfp formed numerous intracellular puncta at the mitochondrion in exponential and stationary-phase yeast cells and was apparent in hyphal cells (Fig. 2B; Fig. S2A and B). Like the prohibitin overexpressing strains, overexpression of Slp2-Gfp did not alter filament structure (Fig. S2D), confirming that only overexpression of Slp3 disrupts filamentation (28). In contrast to the prohibitins, which were dispersed along the entire mitochondrial network, Slp2-Gfp localized throughout the mitochondrial network and at discrete sites as puncta at the mitochondria (compare Fig. 1; Fig. S1; Fig. 2; Fig. S2). The appearance of Slp2-Gfp mitochondrial puncta was consistent with human stomatin orthologs, which also appear as puncta under light microscopy and form membrane microdomains (20, 52, 53). These findings imply that *C. albicans* Slp2 may form mitochondrial membrane complexes.

We were confounded that the expected yellow/orange color change for GFP and MitoTracker Red overlay images was not consistent in *SLP2-GFP*-tagged cells compared to Phb1-Gfp, Phb2-Gfp, and Phb12-Gfp, despite Slp2-Gfp appearing in the same position as the MitoTracker Red-labeled mitochondrion. These Slp2-Gfp puncta appeared as independent green, fluorescent objects in multiple images, suggesting additional sites for Slp2 localization (Fig. 2; Fig. S2). In *S. cerevisiae*, the mitochondrial fission protein Dnm1 and mitochondrial DNA-binding proteins form intracellular puncta, but overlay images do not show an apparent color change for all puncta (54). Although *C. albicans* Slp2 does not contain a known DNA-binding domain, we decided to test for co-localization of Slp2-Gfp with DNA using the DNA-binding dye, NucBlue (Fig. S2C). We also examined the possibility of co-localization between Slp2-Gfp and the vacuole using the vacuolar/endosomal dye, FM 4–64 (Fig. S2C) since *C. albicans* Slp3 was observed inside the vacuole (28). In both cases, Slp2-Gfp did not co-localize with NucBlue or FM 4–64, ruling out possible interactions with DNA or the vacuole (Fig. S2C).

We hypothesized that Slp2 altered the binding of the MitoTracker Red dye to thiol-reactive chloromethyl groups in the mitochondrial membrane (55). Thus, we constructed a *P_TDH3_SLP2-RFP* overexpressing strain and examined co-localization with MitoTracker Green, which binds free thiol groups of cysteine residues of mitochondrial proteins (55). Overlay images showed that overexpressed Slp2-Rfp co-localized with MitoTracker Green in exponential-phase yeast cells (Fig. 2B). Western blotting experiments were performed to confirm Slp2-Gfp mitochondrial co-localization. Immunoblots revealed the presence of a distinct band at approximately 55 kDa, which was the expected size of the Slp2-Gfp fusion protein (Fig. 2C). Collectively, our computational, cytological, and western blot results demonstrate Slp2 localization in *C. albicans* for the first time and reveal that Slp2 mitochondrial localization is conserved.

### SPFH mutant phenotyping

Our cytological results (Fig. 1 and 2; Fig. S1 and S2) and published genetic and biochemical findings from other organisms predict that *C. albicans* Slp2p, Phb1, Phb2, and Phb12 are required for respiration. To test this hypothesis, a panel of homozygous null mutant strains was created using CRISPR-Cas9 mutagenesis (Appendix S1). Double and triple null mutant strains were engineered to assess the possibility of functional redundancy among stomatin and prohibitin proteins.

To determine the extent of stomatin and prohibitin function in respiratory growth, yeast strains were cultured in media containing either 4%–8% glycerol or 2% lactate as non-fermentable carbon sources. Yeast growth was also monitored in media supplemented with the complex III electron transport chain (ETC) inhibitor Antimycin A, the alternate oxidase inhibitor, SHAM, or the cytochrome c oxidase inhibitor SNP. SPFH mutant strains were also exposed to combinatorial treatment with SHAM and SNP. This strategy was shown to examine *C. albicans* mitochondrial function (3) and characterize mitochondrial genes with respiratory functions such as *GOA1* (9). Results of these analyses are summarized in the Appendix S1. In short, we did not observe any respiratory growth defects for any of the conditions examined in our stomatin and prohibitin mutant strains.

Human SLP-2 interacts with prohibitins in the mitochondria to support electron chain assembly, but the exact mechanism is unclear (17). This observation, combined with our mutant phenotypic screening results, suggests a potential compensatory function for stomatins in respiration when prohibitin function is attenuated. Thus, while we cannot completely rule out a role for stomatins and prohibitins in respiration, these genetic results strongly suggest that SPFH proteins are not essential for respiration in *C. albicans* and may have diverged to carry out other mitochondrial functions. Phb1 and Phb2, for example, may instead contribute to protein stability and degradation, as shown in both *S. cerevisiae* and in human fibroblasts (26, 56). Another possibility is that *C. albicans* Phb1 and Phb2 functions may be tied to mitochondrial functions that promote pathogenesis. Indeed, in the fungal plant pathogen, *Colletotrichum higginsianum*, a mitochondrial Phb1/Phb2 complex contributes to virulence via ATG24-assisted mitophagy (57). In addition, another fungal plant pathogen, *Ustilago maydis*, encodes a stomatin protein positively regulated by Gcn5, a histone acetyltransferase whose ortholog in *C. albicans* is known to regulate virulence (58). Despite these findings, we could not find any transcriptomic evidence of altered SPFH gene expression in the context of *in vitro* or *in vivo* infections (59–62).

**Fig 3:**
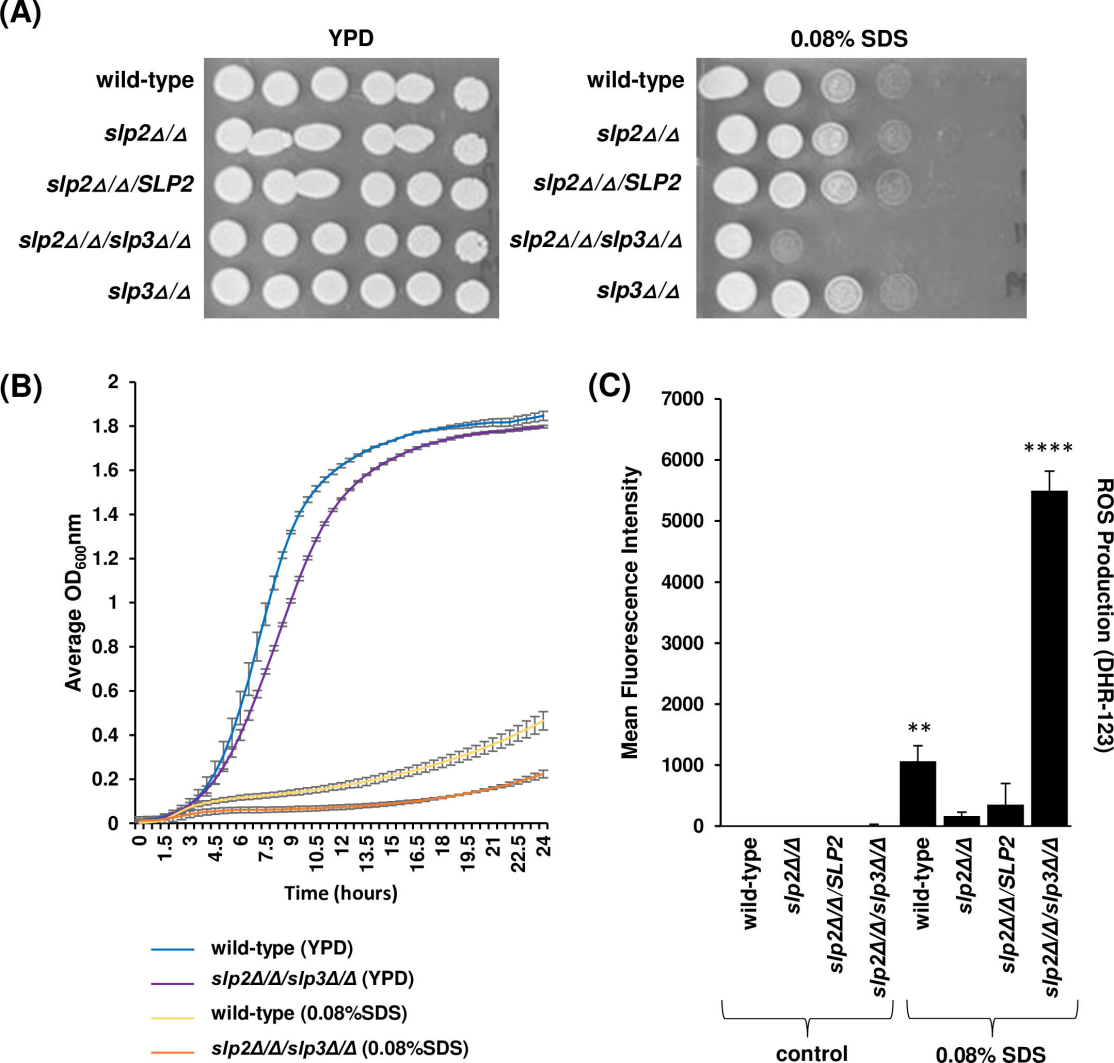
Phenotypes of *C. albicans* stomatin mutants. (**A**) *C. albicans* wild-type, *slp2Δ/Δ, slp2Δ/Δ/SLP2*, *slp2Δ/Δ/slp3Δ/Δ*, and *slp3Δ/Δ* mutant cells were grown on nutrient YPD plates (control) and YPD plates supplemented with 0.08% SDS. Photographs were taken after 48 hours of incubation at 30°C. (**B**) Yeast strain growth was monitored in liquid microplate assays. Approximately 1.0 × 10^4^ cells of each mutant strain and wild-type reference strain were inoculated in YPD medium supplemented with 0.08% SDS or dH_2_O as a control. Cells were grown with shaking at 30°C, and OD_600nm_ readings were collected every 30 minutes. For each assay, three technical replicates were analyzed. Experiments were repeated at least three times, and data presented represent one representative experiment. (**C**) ROS production was measured in stationary-phase wild-type and *slp2Δ/Δ/slp3Δ/Δ* mutant cells following 20 hours of 0.08% SDS treatment or dH_2_O as a control. Yeast cells were incubated with 5 µg/mL DHR-123 and quantified by flow cytometry. For each assay, three technical replicates were analyzed. Experiments were repeated at least three times, and data presented represent one representative experiment. **P < 0.05 and ****P < 0.001 when compared to the isogenic parental strain.

We expanded our phenotypic screens to assess growth under various environmental conditions that require mitochondrial function. Growth for the majority of SPFH mutant strains was similar to the wild-type reference strain when cells were subjected to cation, oxidative, plasma membrane, cell wall, or osmotic stress (Appendix S1). Strikingly, the *slp2Δ*/*Δ*/*slp3Δ*/*Δ* double mutant strain grew poorly in a nutrient medium containing 0.08% SDS in comparison to the wild-type reference and *slp2Δ*/*Δ* and *slp3Δ*/*Δ* single mutant strains (Fig. 3A and B; Appendix S1) (28).

To determine whether hypersensitivity of the *slp2Δ*/*Δ*/*slp3Δ*/*Δ* mutant strain to 0.08% SDS also extended to other surfactants, we also tested its growth in the presence of cetyltrimethylammonium bromide (CTAB), a major cationic surfactant in the topical antiseptic, cetrimide, to determine whether the SDS-sensitive growth phenotype was general to surfactants or specific to SDS. The *slp2Δ*/*Δ*/*slp3Δ*/*Δ* mutant strain grew similar to the wild-type reference strain in the presence of CTAB (Appendix S1). Therefore, Slp2 and Slp3 are both required specifically for adaptation to SDS-mediated cell damage.

SDS inhibits fungal growth in part by increasing the accumulation of intracellular ROS causing oxidative stress (28, 48). ROS levels were evaluated in the *slp2Δ*/*Δ*/*slp3Δ*/*Δ* mutant and wild-type reference strains following 3 and 16 hours of growth in the presence and absence of 0.08% SDS. Flow cytometry results show that 3 and 16 hours of 0.08% SDS treatment significantly increased ROS production in the wild-type reference strain compared to untreated cells (Fig. S3A; Fig. 3C). ROS levels in the *slp2Δ*/*Δ*/*slp3Δ*/*Δ* mutant strain were like the wild-type reference strain in the absence of SDS. On the other hand, ROS levels were up to five times greater in the *slp2Δ*/*Δ*/*slp3Δ*/*Δ* mutant strain treated with 0.08% SDS compared to the wild-type reference strain (Fig. S3A; Fig. 3C). ROS levels in the *slp2Δ*/*Δ* mutant strain were lower than the wild-type strain (Fig. S3A; Fig. 3C). We previously demonstrated that the ROS levels in a *slp3Δ*/*Δ* mutant strain were similar to the wild-type reference strain (28).

The mitochondrial ETC generates ROS, and high intracellular concentrations of ROS cause mitochondrial, nucleic acid, and phospholipid damage, as well as apoptotic-like cell death (63, 64). Because *C. albicans* and human Slp2 reside in the mitochondrion, and human SLP2 associates with ETC components (19, 23), it is possible that the SDS-sensitive *slp2Δ*/*Δ*/*slp3Δ*/*Δ* mutant phenotype may be caused by aberrant ETC function due to loss of *SLP2*. However, we did not observe respiratory or oxidative stress (H_2_O_2_ exposure) phenotypes in the *slp2Δ*/*Δ*, *slp3Δ*/*Δ*, and *slp2Δ*/*Δ*/*slp3Δ*/*Δ* mutant strains (Appendix S1). Also, ROS accumulation in these mutant strains is like the wild-type reference strain in the absence of SDS treatment, refuting this hypothesis. Thus, we argue that the elevated ROS levels in the *slp2Δ*/*Δ*/*slp3Δ*/*Δ* mutant strain are indicative of this strain undergoing severe oxidative stress. However, the molecular basis underlying this *slp2Δ*/*Δ*/*slp3Δ*/*Δ* mutant phenotype is not a consequence of Slp2-dependent ETC mitochondrial dysfunction and suggests other modes of SDS-mediated cell death.

Indeed, a phenotypic screen of an *S. cerevisiae* homozygous diploid deletion library identified 108 SDS-sensitive mutants, of which 79% of the mutant strains accumulated ROS levels greater than the wild-type strain. Notably, apart from the mitochondrial genes *MDM10*, *MDM20*, and 16 uncharacterized genes, the majority of SDS-sensitive mutant strains were not linked to mitochondrial functions. In addition, 21% of mutants had ROS levels similar to or lower than the wild-type strain (48).

To gain further insight into the cellular bases underlying the *slp2Δ*/*Δ*/*slp3Δ*/*Δ* SDS hypersensitive phenotype, we examined the permeability and integrity of the plasma membrane. The lipid-modulating action of detergents and other surfactants is well known. Nevertheless, mounting evidence has shown that SDS-mediated cell damage is not exclusively due to the destruction of the plasma membrane (65). Yeast cells were labeled with the DNA-binding fluorescent viability dye, propidium iodide (PI), following acute (4 hours) and prolonged (16–20 hours) SDS treatment at sub-lethal (0.04%) and lethal (0.08%) concentrations.

PI-labeled cells were not observed following treatment with 0.04% SDS or 4 hours of 0.08% SDS treatment. PI labeling slightly increased for cells treated with 0.08% SDS for 20 hours (Fig. S4). However, there was no difference in the number of PI-labeled cells between the wild-type reference, *slp2Δ*/*Δ*, and *slp2Δ*/*Δ*/*slp3Δ*/*Δ* mutant strains (Fig. S4). This result suggests that altered plasma membrane permeability is not a phenotype specific to the *slp2Δ*/*Δ*/*slp3Δ*/*Δ* mutant strain, but likely a consequence of apoptotic-like cell death caused by prolonged lethal SDS exposure. In *C. albicans*, synthetic surfactant compounds were demonstrated to cause small molecule leakage without causing cell death (65). Also, SDS was shown to kill *C. albicans* without significantly increasing PI labeling or small molecule leakage (66). These observations supported a hypothesis that surfactants may create small pores in the plasma membrane via direct insertion to facilitate entry without directly killing the cell (65).

**Fig 4:**
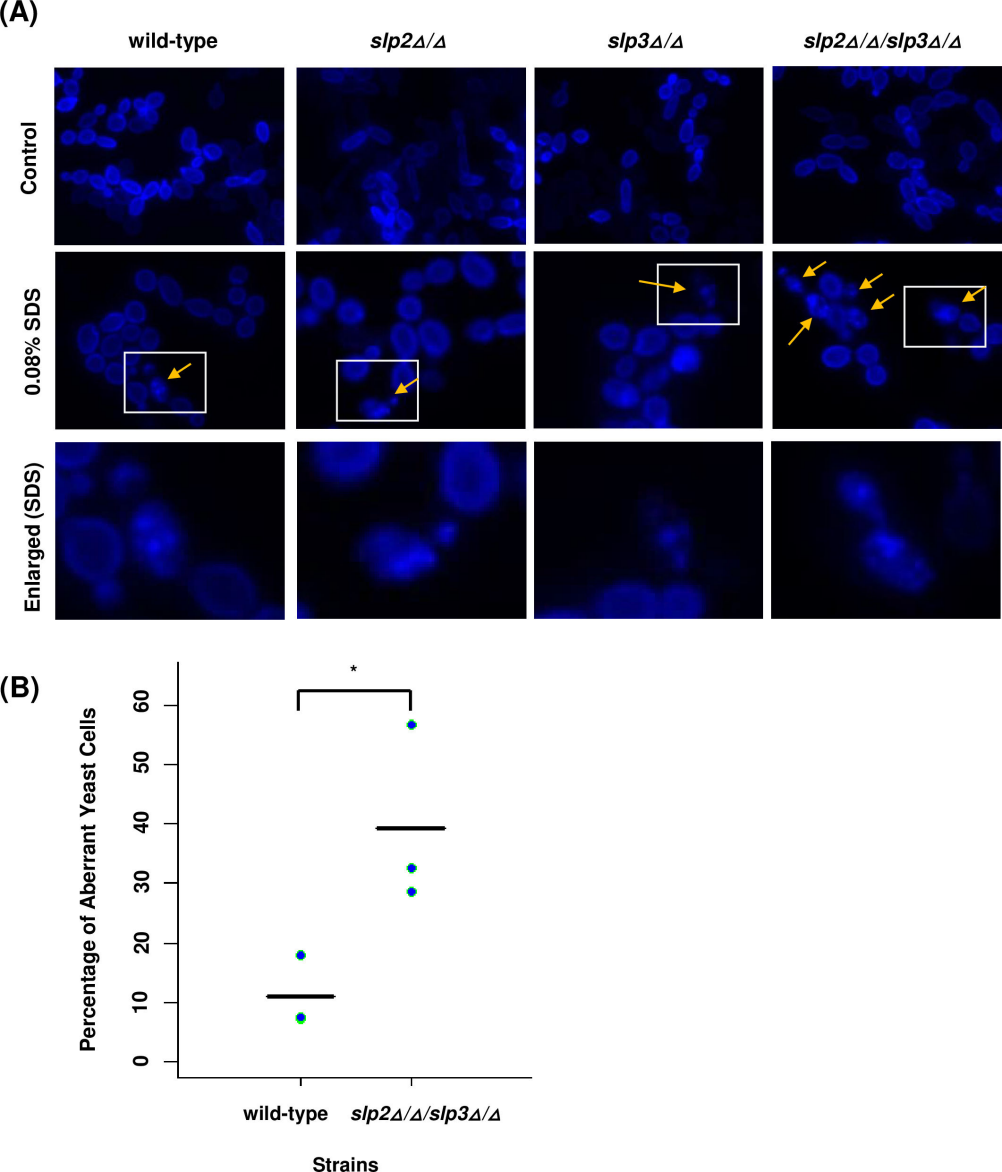
Plasma membrane analysis of *C. albicans* stomatin mutants. (**A**) Plasma membrane ergosterol composition was examined in wild-type, *slp2Δ/Δ, slp3Δ/Δ*, and *slp2Δ/Δ/slp3Δ/Δ* mutant cells following 4 hours of 0.08% SDS treatment or dH_2_O as a control. Cells were labeled with 200 µg/mL filipin and viewed with fluorescent light microscopy. Arrows are used to depict filipin-labeled aberrant yeast cells. The bottom row of pictures (enlarged) depicts 0.08% SDS-treated cells selected in the white box from the images in the middle row. The cells were enlarged to highlight ergosterol labeling. (**B**) Ergosterol plasma membrane distribution was quantified in stationary-phase yeast cells. Filipin-labeled wild-type and *slp2Δ/Δ/slp3Δ/Δ* mutant yeast cells displaying aberrant ergosterol distribution were counted, and the percentage of aberrant cells out of the total number of fluorescent cells (*n* = 132–350 total cells per count) was plotted. Each blue dot represents a single assay, and the black horizontal line represents the average of three experiments. Statistical differences between wild-type and *slp2Δ/Δ/slp3Δ/Δ* mutant yeast cells were determined in paired *t*-tests. *P < 0.05 when compared to the wild-type reference strain.

*C. albicans* strains harboring mutations to the mitochondrial genes *NDH51* and *NUO1* show a drastic reduction in the expression of ergosterol synthetic genes, which directly impacts the composition of the plasma membrane (67). Therefore, we examined the plasma membrane lipid content by labeling cells with the sterol-binding fluorescent dye, filipin. Annular peripheral fluorescence was observed in the *slp2Δ*/*Δ*/*slp3Δ*/*Δ* mutant and wild-type reference strains grown in nutrient media without SDS, showing a uniform distribution of ergosterol in the plasma membrane (Fig. 4A). Following 4 hours of 0.08% SDS treatment, the distribution of ergosterol within the plasma membrane was clearly altered in all strains tested, revealing a mixed population of cells containing ergosterol-rich puncta and cells with normal ergosterol distribution (Fig. 4A). However, aberrant ergosterol distribution was enriched in *slp2Δ*/*Δ*/*slp3Δ*/*Δ* mutant cells compared to wild-type, *slp2Δ*/*Δ*, and *slp3Δ*/*Δ* mutant cells (Fig. 4A). Quantitative analyses of wild-type and *slp2Δ*/*Δ*/*slp3Δ*/*Δ* mutant cells displaying aberrant ergosterol distribution following 4 hours of 0.08% of SDS treatment showed that the percentage of aberrant cells from the *slp2Δ*/*Δ*/*slp3Δ*/*Δ* mutant strain was significantly higher compared to the wild-type reference strain (Fig. 4B). Filipin labeling for all strains was barely detectable after 20 hours of SDS treatment (our unpublished data).

We also examined the *slp2Δ*/*Δ*/*slp3Δ*/*Δ* mutant strain to assess other cellular structures and compartments that are impacted by SDS treatment including the cell wall (68), mitochondria, and intracellular lipid droplets (65). Fluorescent microscopy results did not reveal any apparent defects for these sites in the *slp2Δ*/*Δ*/*slp3Δ*/*Δ* mutant strain in the absence of SDS treatment (Fig. S3B). Following 3 hours of SDS treatment, the amount of lipid droplets increased in all cell types (Fig. S3B) as previously reported for *C. albicans* cells treated with synthetic surfactants (65). SDS treatment increased Calcofluor White labeling (Fig. S3B) which was consistent with published results that demonstrated the impact of SDS exposure on cell wall structure and cell wall damage signaling (68, 69). Lastly, MitoTracker Red labeling increased in all strains; however, mitochondrial structure could not be resolved (Fig. S3B). The basis for this observation is unclear.

Collectively, our cellular results strongly suggest that the loss-of-function of both Slp2 and Slp3 appears to impact ergosterol organization within the plasma membrane. Evidence to support the importance of ergosterol in surfactant tolerance has been derived from genetic analyses in *S. cerevisiae*. Strains containing a null mutation to *ERG3* are highly sensitive to 0.03% SDS (48). *ERG3* encodes a C-5 sterol desaturase, which catalyzes the production of the ergosterol precursor, episterol (70). In addition, the deletion of *ERG3* in *S. cerevisiae* leads to increased tolerance to escin, a mixture of saponin structures that also exhibit surfactant properties (71).

**Fig 5:**
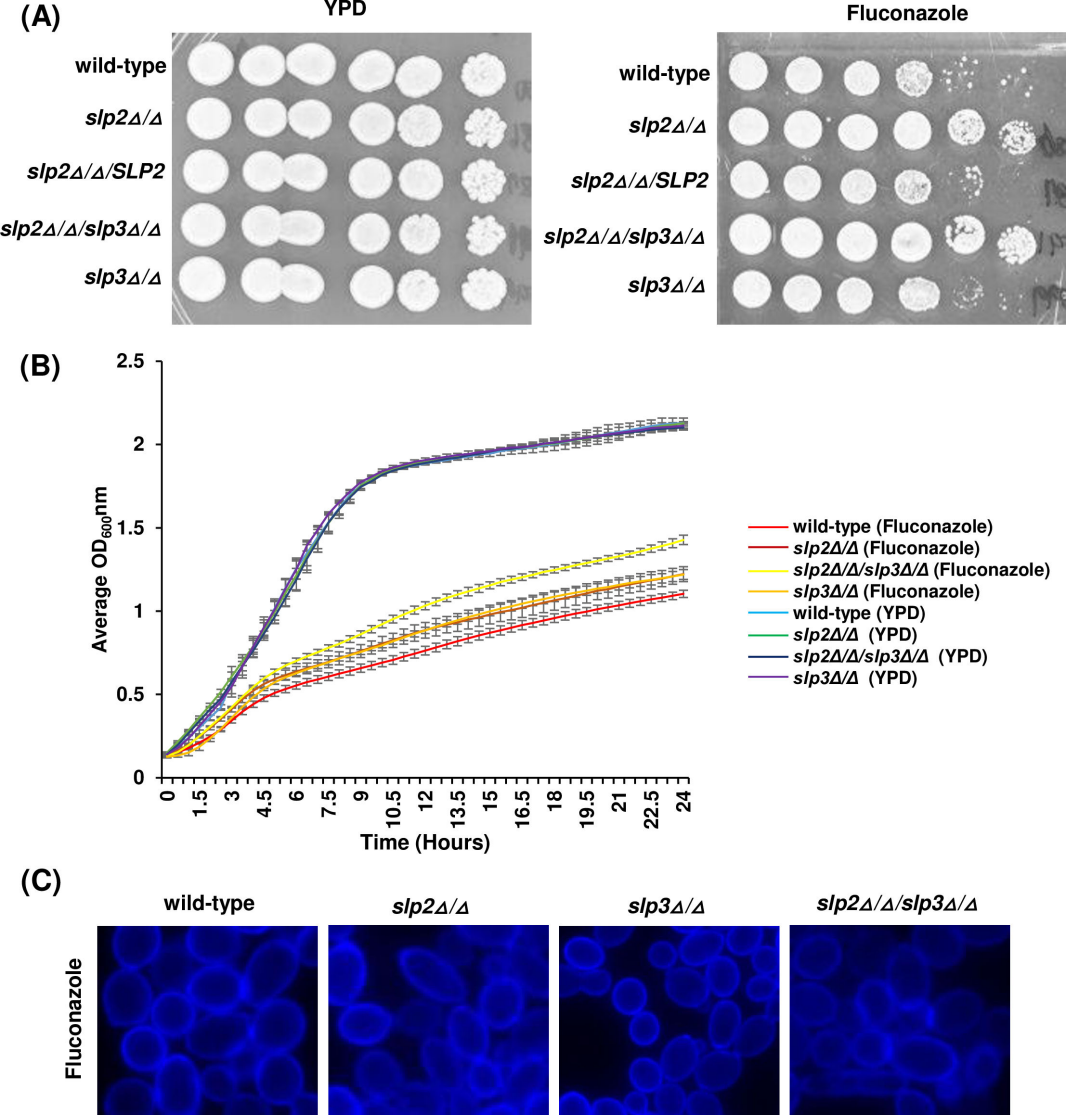
Antifungal resistance of *C. albicans* stomatin mutants. (**A**) *C. albicans* wild-type, *slp2Δ/Δ*, *slp2Δ/Δ/SLP2*, *slp2Δ/Δ/slp3Δ/Δ*, and *slp3Δ/Δ* mutant cells were grown on nutrient YPD plates (control) and YPD plates supplemented with 4.0 µg/mL of fluconazole. Pictures were taken after 48 hours of growth at 30°C. (**B**) Antifungal resistance of *C. albicans* stomatin mutant strains was monitored in YPD liquid cultures (control) and supplemented with 4.0 µg/mL of fluconazole. Cells were grown with shaking at 30°C, and OD_600nm_ readings were collected every 30 minutes. For each assay, three biological replicates were analyzed. Experiments were repeated at least three times, and data presented represent one representative experiment. (**C**) The plasma membrane ergosterol content was examined via filipin labeling in stomatin mutant strains and the wild-type reference strain following 4 hours of incubation in YPD supplemented with 4.0 µg/mL fluconazole.

Surprisingly, the *slp2Δ*/*Δ*/*slp3Δ*/*Δ* and *slp2Δ*/*Δ* mutant strains displayed moderate resistance against the antifungal drug, fluconazole, at concentrations up to 2–4 times that of the wild-type parental strain (Fig. 5A and B; Appendix S1). Specifically, the wild-type parental strain had an MIC of 2 µg/mL, while the *slp2Δ*/*Δ* and *slp2Δ*/*Δ*/*slp3Δ*/*Δ* mutant strains had MIC values of 4 µg/mL, respectively (Appendix S1). However, the *slp3Δ*/*Δ* mutant strain remained susceptible to fluconazole at levels similar to the wild-type reference strain (Fig. 5A and B; Appendix S1) (28). We did not observe aberrant ergosterol organization following 4 hours of fluconazole treatment as observed with SDS-treated cells (compare Fig. 4A and 5C). Therefore, these findings associate stomatins with antifungal resistance for the first time.

Given our findings herein, we propose that Slp2 and Slp3 indirectly interact to coordinate SDS tolerance by stabilizing lipid dynamics between the plasma membrane, vacuole (the target sites of Slp3), and mitochondria (the target site of Slp2). This interaction may occur at the post-translational level in *C. albicans* because *SLP2* transcription is not modulated in a *slp3Δ*/*Δ* mutant strain (28). This hypothesis is supported by the following lines of evidence: Human stomatin (STOM) binds to cholesterol, and murine cells overexpressing STOM showed altered fatty acid uptake and enlarged lipid droplets (20, 72). Also, human STOM interacts with SLP-1 in lipid trafficking between the plasma membrane and late endosome (73). In *S. cerevisiae*, yeast strains containing mutations to genes involved in vacuolar transport and function and ergosterol synthesis are highly sensitive to SDS (48). The widespread aberrant distribution of ergosterol in the plasma membrane following SDS exposure in the *slp2Δ*/*Δ*/*slp3Δ*/*Δ* mutant strain suggests that Slp2 and Slp3 coordinate ergosterol trafficking and organization in *C. albicans*. Lastly, the fluconazole-resistant phenotype of the *slp2Δ*/*Δ*/*slp3Δ*/*Δ* mutant strain further links *C. albicans* stomatins to ergosterol synthesis and organization.

### Conclusion

The ubiquitous presence of SPFH proteins in virtually all membranes underscores their biological importance in the tree of life, yet an understanding of their molecular function remains elusive. Here, we show that *C. albicans* SPFH protein mitochondrial localization is conserved, but the molecular function for these proteins has apparently diverged toward non-respiratory roles. Moreover, our findings with Slp2 and Slp3 implicate potential interactions to coordinate plasma membrane and mitochondrion dynamics in response to SDS-induced cellular damage. While the molecular mechanisms underlying fluconazole resistance and SDS sensitivity in the *slp2Δ*/*Δ*/*slp3Δ*/*Δ* mutant strain are unknown, the stomatin mitochondrial-plasma membrane connection must be considered. Characterization of the type and mode of interactions between Slp2 and Slp3 warrants further investigation. Proteomic analyses, such as liquid chromatography-mass spectrometry, paired with the genetic and cellular analyses used herein, may identify direct prohibitin and stomatin binding targets and provide molecular mechanistic insight into *C. albicans* SPFH protein function.

## SUPPLEMENTAL MATERIAL

Figure S1 spectrum.01733-24-s0001.tif Prohibitin localization in yeast and hyphal cells.

Figure S2 spectrum.01733-24-s0002.tif Slp2 localization in yeast and hyphal cells.

Figure S3 spectrum.01733-24-s0003.tif Mitochondrial and cytological analyses of stomatin mutants.

Figure S4 spectrum.01733-24-s0004.tif Analysis of plasma membrane permeability in stomatin mutants.

Supplemental appendix spectrum.01733-24-s0005.xlsx Includes yeast strain genotypes, oligonucleotide sequences, and results of phenotypic growth assays in tabular format.

Supplemental material spectrum.01733-24-s0006.docx Supplemental figure and appendix legends.

ASM does not own the copyrights to Supplemental Material that may be linked to, or accessed through, an article. The authors have granted ASM a non-exclusive, world-wide license to publish the Supplemental Material files. Please contact the corresponding author directly for reuse.

## References

[1] Richard A, Calderone CJC, eds. 2012. Candida and candidiasis. ASM Press, Washington, DC.

[2] Sun N, Parrish RS, Calderone RA, Fonzi WA. 2019. Unique, diverged, and conserved mitochondrial functions influencing Candida albicans respiration. mBio 10:e00300-19. doi:10.1128/mBio.00300-19 31239372 PMC6593398

[3] Duvenage L, Walker LA, Bojarczuk A, Johnston SA, MacCallum DM, Munro CA, Gourlay CW. 2019. Inhibition of classical and alternative modes of respiration in Candida albicans leads to cell wall remodeling and increased macrophage recognition. mBio 10:e02535-18. doi:10.1128/mBio.02535-18 30696734 PMC6355986

[4] Koch B, Tucey TM, Lo TL, Novakovic S, Boag P, Traven A. 2017. The mitochondrial GTPase Gem1 contributes to the cell wall stress response and invasive growth of Candida albicans. Front Microbiol 8:2555. doi:10.3389/fmicb.2017.02555 29326680 PMC5742345

[5] Calderone R, Li D, Traven A. 2015. System-level impact of mitochondria on fungal virulence: to metabolism and beyond. FEMS Yeast Res 15:fov027. doi:10.1093/femsyr/fov027 26002841 PMC4542695

[6] Mamouei Z, Singh S, Lemire B, Gu Y, Alqarihi A, Nabeela S, Li D, Ibrahim A, Uppuluri P. 2021. An evolutionarily diverged mitochondrial protein controls biofilm growth and virulence in Candida albicans. PLoS Biol 19:e3000957. doi:10.1371/journal.pbio.3000957 33720927 PMC8007014

[7] Benhamou RI, Bibi M, Steinbuch KB, Engel H, Levin M, Roichman Y, Berman J, Fridman M. 2017. Real-time imaging of the azole class of antifungal drugs in live Candida cells. ACS Chem Biol 12:1769–1777. doi:10.1021/acschembio.7b00339 28472585 PMC7030953

[8] She X, Khamooshi K, Gao Y, Shen Y, Lv Y, Calderone R, Fonzi W, Liu W, Li D. 2015. Fungal-specific subunits of the Candida albicans mitochondrial complex I drive diverse cell functions including cell wall synthesis. Cell Microbiol 17:1350–1364. doi:10.1111/cmi.12438 25801605 PMC4677794

[9] Bambach A, Fernandes MP, Ghosh A, Kruppa M, Alex D, Li D, Fonzi WA, Chauhan N, Sun N, Agrellos OA, Vercesi AE, Rolfes RJ, Calderone R. 2009. Goa1p of Candida albicans localizes to the mitochondria during stress and is required for mitochondrial function and virulence. Eukaryot Cell 8:1706–1720. doi:10.1128/EC.00066-09 19717740 PMC2772395

[10] Ma C, Wang C, Luo D, Yan L, Yang W, Li N, Gao N. 2022. Structural insights into the membrane microdomain organization by SPFH family proteins. Cell Res 32:176–189. doi:10.1038/s41422-021-00598-3 34975153 PMC8807802

[11] Heredia MY, Rauceo JM. 2021. The SPFH protein superfamily in fungi: impact on mitochondrial function and implications in virulence. Microorganisms 9:2287. doi:10.3390/microorganisms9112287 34835412 PMC8624314

[12] Lapatsina L, Brand J, Poole K, Daumke O, Lewin GR. 2012. Stomatin-domain proteins. Eur J Cell Biol 91:240–245. doi:10.1016/j.ejcb.2011.01.018 21501885

[13] Wetzel C, Hu J, Riethmacher D, Benckendorff A, Harder L, Eilers A, Moshourab R, Kozlenkov A, Labuz D, Caspani O, Erdmann B, Machelska H, Heppenstall PA, Lewin GR. 2007. A stomatin-domain protein essential for touch sensation in the mouse. Nature 445:206–209. doi:10.1038/nature05394 17167420

[14] Chowdhury I, Thompson WE, Thomas K. 2014. A stomatin-domain protein essential for touch sensation in the mouse. J Cell Physiol 229:998–1004. doi:10.1002/jcp.24531 24347342 PMC4413917

[15] Liu Q, Yao F, Jiang G, Xu M, Chen S, Zhou L, Sakamoto N, Kuno T, Fang Y. 2018. Dysfunction of prohibitin 2 results in reduced susceptibility to multiple antifungal drugs via activation of the oxidative stress-responsive transcription factor Pap1 in fission yeast. Antimicrob Agents Chemother 62:e00860-18. doi:10.1128/AAC.00860-18 30181366 PMC6201106

[16] Rungaldier S, Oberwagner W, Salzer U, Csaszar E, Prohaska R. 2013. Stomatin interacts with GLUT1/SLC2A1, band 3/SLC4A1, and aquaporin-1 in human erythrocyte membrane domains. Biochim Biophys Acta 1828:956–966. doi:10.1016/j.bbamem.2012.11.030 23219802 PMC3790964

[17] Da Cruz S, Parone PA, Gonzalo P, Bienvenut WV, Tondera D, Jourdain A, Quadroni M, Martinou J-C. 2008. SLP-2 interacts with prohibitins in the mitochondrial inner membrane and contributes to their stability. Biochim Biophys Acta 1783:904–911. doi:10.1016/j.bbamcr.2008.02.006 18339324

[18] Osman C, Wilmes C, Tatsuta T, Langer T. 2007. Prohibitins interact genetically with Atp23, a novel processing peptidase and chaperone for the F1Fo-ATP synthase. Mol Biol Cell 18:627–635. doi:10.1091/mbc.e06-09-0839 17135288 PMC1783772

[19] Christie DA, Lemke CD, Elias IM, Chau LA, Kirchhof MG, Li B, Ball EH, Dunn SD, Hatch GM, Madrenas J. 2011. Stomatin-like protein 2 binds cardiolipin and regulates mitochondrial biogenesis and function. Mol Cell Biol 31:3845–3856. doi:10.1128/MCB.05393-11 21746876 PMC3165718

[20] Wu SC, Lo YM, Lee JH, Chen CY, Chen TW, Liu HW, Lian WN, Hua K, Liao CC, Lin WJ, Yang CY, Tung CY, Lin CH. 2022. Stomatin modulates adipogenesis through the ERK pathway and regulates fatty acid uptake and lipid droplet growth. Nat Commun 13:4174. doi:10.1038/s41467-022-31825-z 35854007 PMC9296665

[21] Signorile A, Sgaramella G, Bellomo F, De Rasmo D. 2019. Prohibitins: a critical role in mitochondrial functions and implication in diseases. Cells 8:71. doi:10.3390/cells8010071 30669391 PMC6356732

[22] Wei Y, Chiang WC, Sumpter Jr R, Mishra P, Levine B. 2017. Prohibitin 2 is an inner mitochondrial membrane mitophagy receptor. Cell 168:224–238. doi:10.1016/j.cell.2016.11.042 28017329 PMC5235968

[23] Wai T, Saita S, Nolte H, Müller S, König T, Richter-Dennerlein R, Sprenger H-G, Madrenas J, Mühlmeister M, Brandt U, Krüger M, Langer T. 2016. The membrane scaffold SLP2 anchors a proteolytic hub in mitochondria containing PARL and the i‐AAA protease YME1L. EMBO Rep 17:1844–1856. doi:10.15252/embr.201642698 27737933 PMC5283581

[24] Matz JM, Goosmann C, Matuschewski K, Kooij TWA. 2018. An unusual prohibitin regulates malaria parasite mitochondrial membrane potential. Cell Rep 23:756–767. doi:10.1016/j.celrep.2018.03.088 29669282

[25] Tatsuta T, Model K, Langer T. 2005. Formation of membrane-bound ring complexes by prohibitins in mitochondria. Mol Biol Cell 16:248–259. doi:10.1091/mbc.e04-09-0807 15525670 PMC539169

[26] Nijtmans LG, de Jong L, Artal Sanz M, Coates PJ, Berden JA, Back JW, Muijsers AO, van der Spek H, Grivell LA. 2000. Prohibitins act as a membrane-bound chaperone for the stabilization of mitochondrial proteins. EMBO J 19:2444–2451. doi:10.1093/emboj/19.11.2444 10835343 PMC212747

[27] Berger KH, Yaffe MP. 1998. Prohibitin family members interact genetically with mitochondrial inheritance components in Saccharomyces cerevisiae. Mol Cell Biol 18:4043–4052. doi:10.1128/MCB.18.7.4043 9632789 PMC108989

[28] Conrad KA, Rodriguez R, Salcedo EC, Rauceo JM. 2018. The Candida albicans stress response gene stomatin-like protein 3 is implicated in ROS-induced apoptotic-like death of yeast phase cells. PLoS One 13:e0192250. doi:10.1371/journal.pone.0192250 29389961 PMC5794166

[29] Marotta DH, Nantel A, Sukala L, Teubl JR, Rauceo JM. 2013. Genome-wide transcriptional profiling and enrichment mapping reveal divergent and conserved roles of Sko1 in the Candida albicans osmotic stress response. Genomics 102:363–371. doi:10.1016/j.ygeno.2013.06.002 23773966 PMC3907168

[30] Chaves GM, da Silva WP. 2012. Superoxide dismutases and glutaredoxins have a distinct role in the response of Candida albicans to oxidative stress generated by the chemical compounds menadione and diamide. Mem Inst Oswaldo Cruz 107:998–1005. doi:10.1590/s0074-02762012000800006 23295749

[31] Martchenko M, Alarco AM, Harcus D, Whiteway M. 2004. Superoxide dismutases in Candida albicans: transcriptional regulation and functional characterization of the hyphal-induced SOD5 gene. Mol Biol Cell 15:456–467. doi:10.1091/mbc.e03-03-0179 14617819 PMC329211

[32] Conrad KA, Kim H, Qasim M, Djehal A, Hernday AD, Désaubry L, Rauceo JM. 2023. Triazine-based small molecules: a potential new class of compounds in the antifungal toolbox. Pathogens 12:126. doi:10.3390/pathogens12010126 36678474 PMC9861074

[33] Walther A, Wendland J. 2003. An improved transformation protocol for the human fungal pathogen Candida albicans. Curr Genet 42:339–343. doi:10.1007/s00294-002-0349-0 12612807

[34] Magee PT, Gale C, Berman J, Davis D. 2003. Molecular genetic and genomic approaches to the study of medically important fungi. Infect Immun 71:2299–2309. doi:10.1128/IAI.71.5.2299-2309.2003 12704098 PMC153231

[35] Gerami-Nejad M, Berman J, Gale CA. 2001. Cassettes for PCR-mediated construction of green, yellow, and cyan fluorescent protein fusions in Candida albicans. Yeast 18:859–864. doi:10.1002/yea.738 11427968

[36] Gerami-Nejad M, Dulmage K, Berman J. 2009. Additional cassettes for epitope and fluorescent fusion proteins in Candida albicans. Yeast 26:399–406. doi:10.1002/yea.1674 19504625 PMC3086567

[37] Heredia MY, Ikeh MAC, Gunasekaran D, Conrad KA, Filimonava S, Marotta DH, Nobile CJ, Rauceo JM. 2020. An expanded cell wall damage signaling network is comprised of the transcription factors Rlm1 and Sko1 in Candida albicans. PLoS Genet 16:e1008908. doi:10.1371/journal.pgen.1008908 32639995 PMC7371209

[38] Seher TD, Nguyen N, Ramos D, Bapat P, Nobile CJ, Sindi SS, Hernday AD. 2021. AddTag, a two-step approach with supporting software package that facilitates CRISPR/Cas-mediated precision genome editing. G3 (Bethesda) 11:jkab216. doi:10.1093/g3journal/jkab216 34544122 PMC8496238

[39] David C. Amberg DJB, Strathern JN. 2005. Edited by J. Inglis. Methods in yeast genetics. New York.

[40] Rauceo JM, Blankenship JR, Fanning S, Hamaker JJ, Deneault J-S, Smith FJ, Nantel A, Mitchell AP. 2008. Regulation of the Candida albicans cell wall damage response by transcription factor Sko1 and PAS kinase Psk1. Mol Biol Cell 19:2741–2751. doi:10.1091/mbc.e08-02-0191 18434592 PMC2441657

[41] Jones P, Binns D, Chang HY, Fraser M, Li W, McAnulla C, McWilliam H, Maslen J, Mitchell A, Nuka G, Pesseat S, Quinn AF, Sangrador-Vegas A, Scheremetjew M, Yong SY, Lopez R, Hunter S. 2014. InterProScan 5: genome-scale protein function classification. Bioinformatics 30:1236–1240. doi:10.1093/bioinformatics/btu031 24451626 PMC3998142

[42] Fukasawa Y, Tsuji J, Fu SC, Tomii K, Horton P, Imai K. 2015. MitoFates: improved prediction of mitochondrial targeting sequences and their cleavage sites. Mol Cell Proteomics 14:1113–1126. doi:10.1074/mcp.M114.043083 25670805 PMC4390256

[43] Indio V, Martelli PL, Savojardo C, Fariselli P, Casadio R. 2013. The prediction of organelle-targeting peptides in eukaryotic proteins with grammatical-restrained hidden conditional random fields. Bioinformatics 29:981–988. doi:10.1093/bioinformatics/btt089 23428638

[44] Liu W, Xie Y, Ma J, Luo X, Nie P, Zuo Z, Lahrmann U, Zhao Q, Zheng Y, Zhao Y, Xue Y, Ren J. 2015. IBS: an illustrator for the presentation and visualization of biological sequences. Bioinformatics 31:3359–3361. doi:10.1093/bioinformatics/btv362 26069263 PMC4595897

[45] Skrzypek MS, Binkley J, Binkley G, Miyasato SR, Simison M, Sherlock G. 2017. The Candida Genome Database (CGD): incorporation of assembly 22, systematic identifiers and visualization of high throughput sequencing data. Nucleic Acids Res 45:D592–D596. doi:10.1093/nar/gkw924 27738138 PMC5210628

[46] Uppuluri P, Chaffin WL. 2007. Defining Candida albicans stationary phase by cellular and DNA replication, gene expression and regulation. Mol Microbiol 64:1572–1586. doi:10.1111/j.1365-2958.2007.05760.x 17555439

[47] Kaloriti D, Jacobsen M, Yin Z, Patterson M, Tillmann A, Smith DA, Cook E, You T, Grimm MJ, Bohovych I, Grebogi C, Segal BH, Gow NAR, Haynes K, Quinn J, Brown AJP. 2014. Mechanisms underlying the exquisite sensitivity of Candida albicans to combinatorial cationic and oxidative stress that enhances the potent fungicidal activity of phagocytes. mBio 5:e01334-14. doi:10.1128/mBio.01334-14 25028425 PMC4161263

[48] Cao C, Cao Z, Yu P, Zhao Y. 2020. Genome-wide identification for genes involved in sodium dodecyl sulfate toxicity in Saccharomyces cerevisiae. BMC Microbiol 20:34. doi:10.1186/s12866-020-1721-2 32066383 PMC7027087

[49] Heredia MY, Gunasekaran D, Ikeh MAC, Nobile CJ, Rauceo JM. 2020. Transcriptional regulation of the caspofungin-induced cell wall damage response in Candida albicans. Curr Genet 66:1059–1068. doi:10.1007/s00294-020-01105-8 32876716 PMC7724954

[50] Vasicek EM, Berkow EL, Bruno VM, Mitchell AP, Wiederhold NP, Barker KS, Rogers PD. 2014. Disruption of the transcriptional regulator Cas5 results in enhanced killing of Candida albicans by fluconazole. Antimicrob Agents Chemother 58:6807–6818. doi:10.1128/AAC.00064-14 25182640 PMC4249418

[51] Nantel A, Dignard D, Bachewich C, Harcus D, Marcil A, Bouin A-P, Sensen CW, Hogues H, van het Hoog M, Gordon P, Rigby T, Benoit F, Tessier DC, Thomas DY, Whiteway M. 2002. Transcription profiling of Candida albicans cells undergoing the yeast-to-hyphal transition. Mol Biol Cell 13:3452–3465. doi:10.1091/mbc.e02-05-0272 12388749 PMC129958

[52] Snyers L, Umlauf E, Prohaska R. 1998. Oligomeric nature of the integral membrane protein stomatin. J Biol Chem 273:17221–17226. doi:10.1074/jbc.273.27.17221 9642292

[53] Christie DA, Kirchhof MG, Vardhana S, Dustin ML, Madrenas J. 2012. Mitochondrial and plasma membrane pools of stomatin-like protein 2 coalesce at the immunological synapse during T cell activation. PLoS One 7:e37144. doi:10.1371/journal.pone.0037144 22623988 PMC3356372

[54] Klecker T, Wemmer M, Haag M, Weig A, Böckler S, Langer T, Nunnari J, Westermann B. 2015. Interaction of MDM33 with mitochondrial inner membrane homeostasis pathways in yeast. Sci Rep 5:18344. doi:10.1038/srep18344 26669658 PMC4680886

[55] Clutton G, Mollan K, Hudgens M, Goonetilleke N. 2019. A reproducible, objective method using MitoTracker® fluorescent dyes to assess mitochondrial mass in T cells by flow cytometry. Cyto A 95:450–456. doi:10.1002/cyto.a.23705 PMC6461488 30576071

[56] Steglich G, Neupert W, Langer T. 1999. Prohibitins regulate membrane protein degradation by the m-AAA protease in mitochondria. Mol Cell Biol 19:3435–3442. doi:10.1128/MCB.19.5.3435 10207067 PMC84136

[57] Yan Y, Tang J, Yuan Q, Liu C, Chen X, Liu H, Huang J, Bao C, Hsiang T, Zheng L. 2022. Mitochondrial prohibitin complex regulates fungal virulence via ATG24-assisted mitophagy. Commun Biol 5:698. doi:10.1038/s42003-022-03666-5 35835849 PMC9283515

[58] Martínez-Soto D, González-Prieto JM, Ruiz-Herrera J. 2015. Transcriptomic analysis of the GCN5 gene reveals mechanisms of the epigenetic regulation of virulence and morphogenesis in Ustilago maydis. FEMS Yeast Res 15:fov055. doi:10.1093/femsyr/fov055 26126523

[59] Walker LA, Maccallum DM, Bertram G, Gow NAR, Odds FC, Brown AJP. 2009. Genome-wide analysis of Candida albicans gene expression patterns during infection of the mammalian kidney. Fungal Genet Biol 46:210–219. doi:10.1016/j.fgb.2008.10.012 19032986 PMC2698078

[60] Muñoz JF, Delorey T, Ford CB, Li BY, Thompson DA, Rao RP, Cuomo CA. 2019. Coordinated host-pathogen transcriptional dynamics revealed using sorted subpopulations and single macrophages infected with Candida albicans. Nat Commun 10:1607. doi:10.1038/s41467-019-09599-8 30962448 PMC6453965

[61] Bruno VM, Shetty AC, Yano J, Fidel PL, Noverr MC, Peters BM. 2015. Transcriptomic analysis of vulvovaginal candidiasis identifies a role for the NLRP3 inflammasome. mBio 6:e00182-15. doi:10.1128/mBio.00182-15 25900651 PMC4453569

[62] Liu Y, Shetty AC, Schwartz JA, Bradford LL, Xu W, Phan QT, Kumari P, Mahurkar A, Mitchell AP, Ravel J, Fraser CM, Filler SG, Bruno VM. 2015. New signaling pathways govern the host response to C. albicans infection in various niches. Genome Res 25:679–689. doi:10.1101/gr.187427.114 25858952 PMC4417116

[63] Madeo F, Fröhlich E, Ligr M, Grey M, Sigrist SJ, Wolf DH, Fröhlich KU. 1999. Oxygen stress: a regulator of apoptosis in yeast. J Cell Biol 145:757–767. doi:10.1083/jcb.145.4.757 10330404 PMC2133192

[64] Fleury C, Mignotte B, Vayssière J-L. 2002. Mitochondrial reactive oxygen species in cell death signaling. Biochimie 84:131–141. doi:10.1016/s0300-9084(02)01369-x 12022944

[65] Paluch E, Szperlik J, Lamch Ł, Wilk KA, Obłąk E. 2021. Biofilm eradication and antifungal mechanism of action against Candida albicans of cationic dicephalic surfactants with a labile linker. Sci Rep 11:8896. doi:10.1038/s41598-021-88244-1 33903615 PMC8076202

[66] Yu Q, Zhang B, Ma F, Jia C, Xiao C, Zhang B, Xing L, Li M. 2015. Novel mechanisms of surfactants against Candida albicans growth and morphogenesis. Chem Biol Interact 227:1–6. doi:10.1016/j.cbi.2014.12.014 25523088

[67] Li D, She X, Calderone R. 2016. Functional diversity of complex I subunits in Candida albicans mitochondria. Curr Genet 62:87–95. doi:10.1007/s00294-015-0518-6 26373419 PMC4724564

[68] Schroeder L, Ikui AE. 2019. Tryptophan confers resistance to SDS-associated cell membrane stress in Saccharomyces cerevisiae. PLoS One 14:e0199484. doi:10.1371/journal.pone.0199484 30856175 PMC6411118

[69] Bruno VM, Kalachikov S, Subaran R, Nobile CJ, Kyratsous C, Mitchell AP. 2006. Control of the C. albicans cell wall damage response by transcriptional regulator Cas5. PLoS Pathog 2:e21. doi:10.1371/journal.ppat.0020021 16552442 PMC1401495

[70] Lees ND, Skaggs B, Kirsch DR, Bard M. 1995. Cloning of the late genes in the ergosterol biosynthetic pathway of Saccharomyces cerevisiae—A review. Lipids 30:221–226. doi:10.1007/BF02537824 7791529

[71] Johnston EJ, Tallis J, Cunningham-Oakes E, Moses T, Moore SJ, Hosking S, Rosser SJ. 2023. Yeast lacking the sterol C-5 desaturase Erg3 are tolerant to the anti-inflammatory triterpenoid saponin escin. Sci Rep 13:13617. doi:10.1038/s41598-023-40308-0 37604855 PMC10442444

[72] Rungaldier S, Umlauf E, Mairhofer M, Salzer U, Thiele C, Prohaska R. 2017. Structure-function analysis of human stomatin: a mutation study. PLoS One 12:e0178646. doi:10.1371/journal.pone.0178646 28575093 PMC5456319

[73] Mairhofer M, Steiner M, Salzer U, Prohaska R. 2009. Stomatin-like protein-1 interacts with stomatin and is targeted to late endosomes. J Biol Chem 284:29218–29229. doi:10.1074/jbc.M109.014993 19696025 PMC2781465

