# Supplementary figures and images for: Mitochondrial targeting of Candida albicans SPFH proteins and requirement of stomatins for SDS-induced stress tolerance
Source: Microbiol Spectr. 2024 Dec 6;13(1):e01733-24. doi: 10.1128/spectrum.01733-24 (PMC11705831; doi:10.1128/spectrum.01733-24)

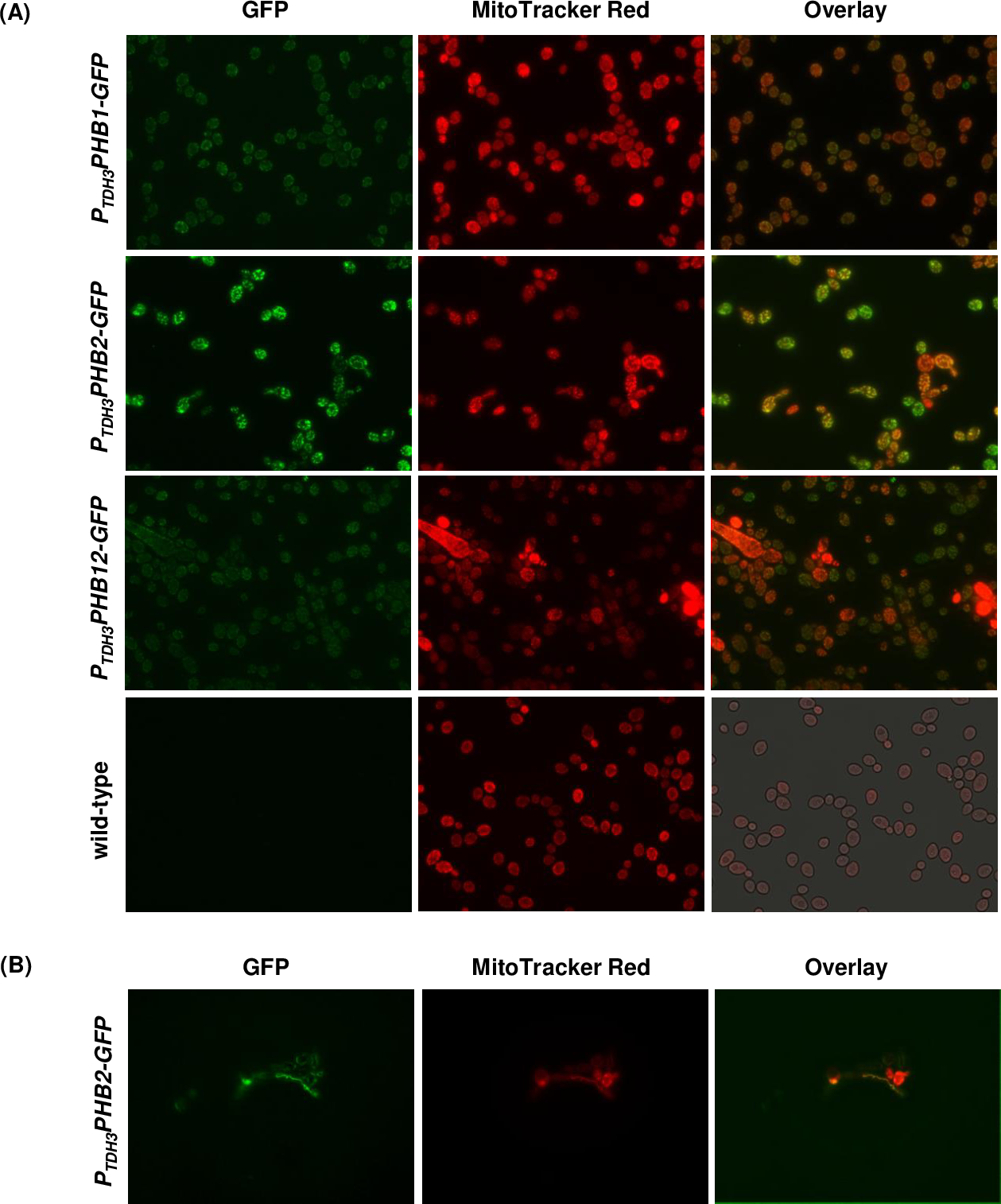

Supplement: Figure S1 — Prohibitin localization in yeast and hyphal cells. [file spectrum.01733-24-s0001.tif]

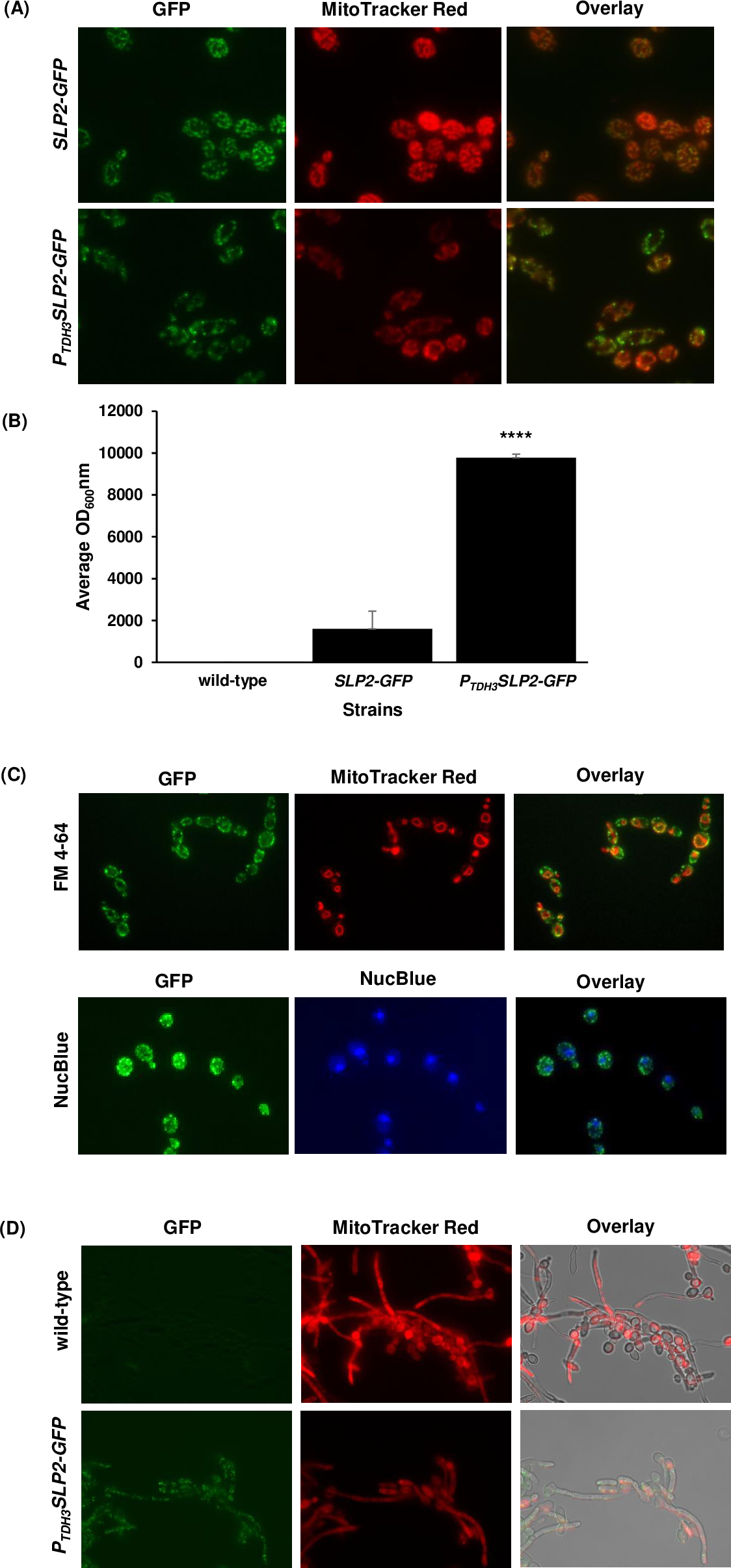

Supplement: Figure S2 — Slp2 localization in yeast and hyphal cells. [file spectrum.01733-24-s0002.tif]

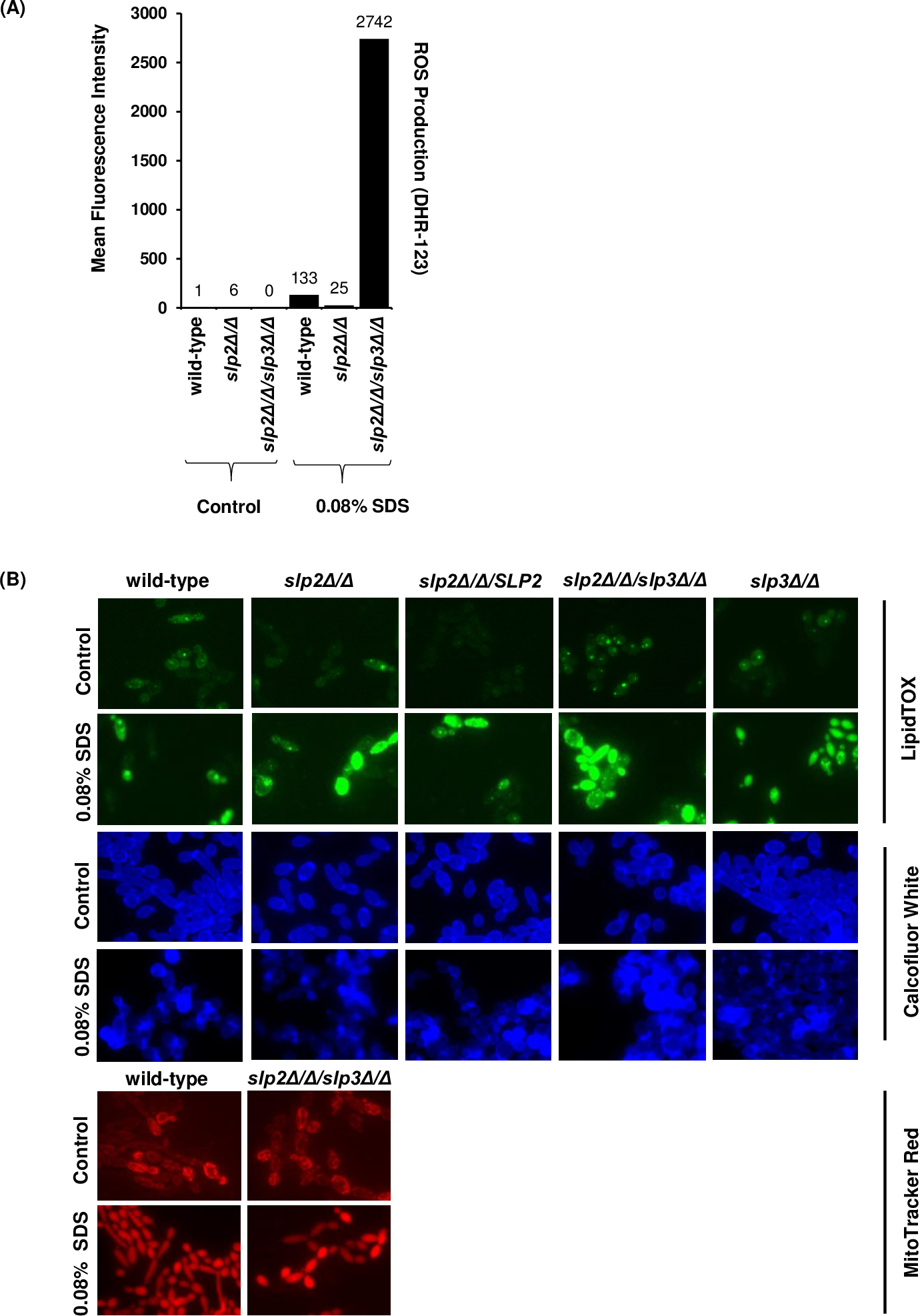

Supplement: Figure S3 — Mitochondrial and cytological analyses of stomatin mutants. [file spectrum.01733-24-s0003.tif]

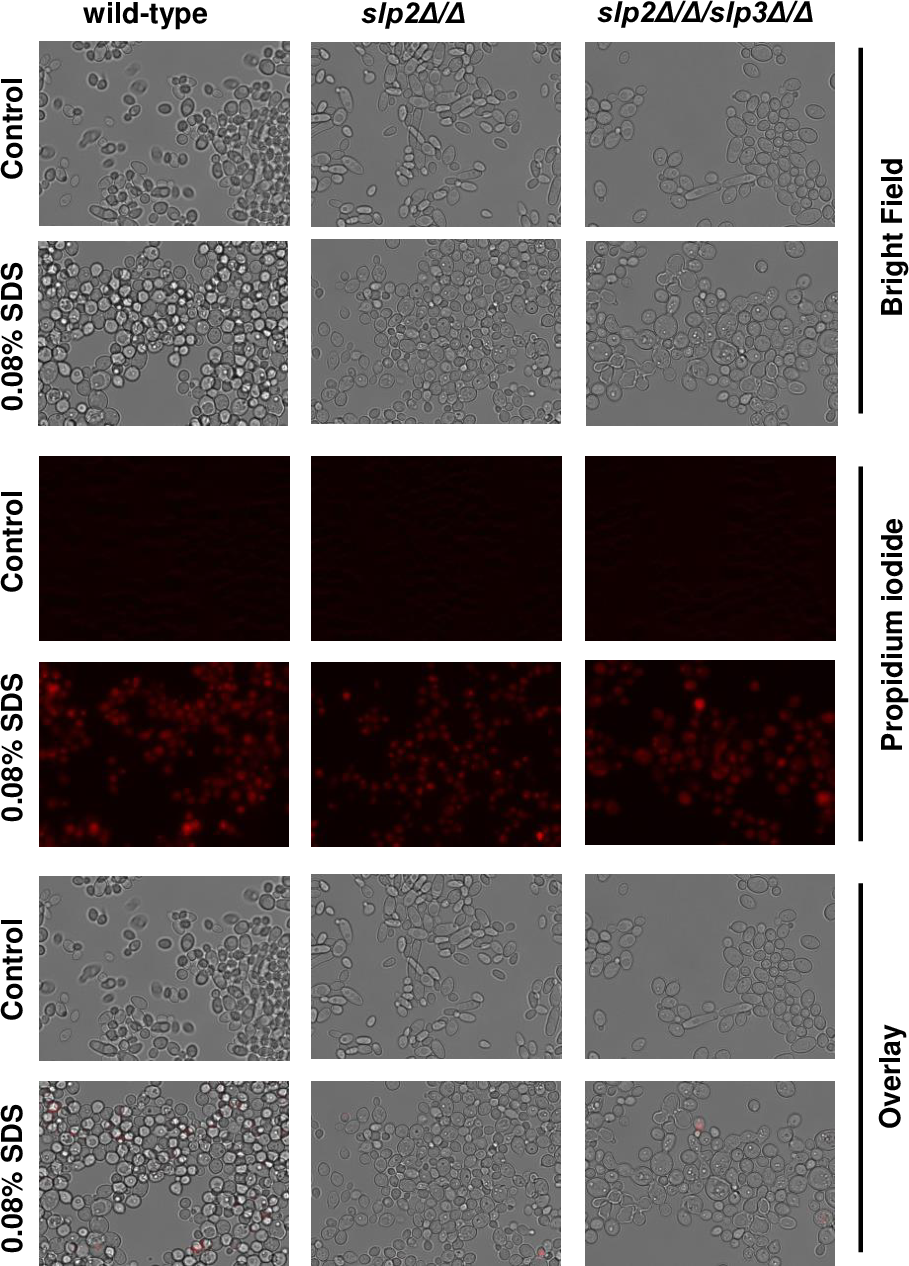

Supplement: Figure S4 — Analysis of plasma membrane permeability in stomatin mutants. [file spectrum.01733-24-s0004.tif]
