# Supplementary material for: Mitochondrial targeting of Candida albicans SPFH proteins and requirement of stomatins for SDS-induced stress tolerance
Source: Microbiol Spectr. 2024 Dec 6;13(1):e01733-24. doi: 10.1128/spectrum.01733-24 (PMC11705831; doi:10.1128/spectrum.01733-24)
Supplement: Supplemental material — Supplemental figure and appendix legends. [file spectrum.01733-24-s0006.docx]

**Supplemental Figures**

**FIG S1. Prohibitin localization in yeast and hyphal cells.** (A) Stationary-phase *P_TDH3_PHB1-GFP*, *P_TDH3_PHB2-GFP* and *P_TDH3_PHB12-GFP* over-expressing cells were observed using fluorescent microscopy, and prohibitin localization at the mitochondria was examined in MitoTracker Red labeled cells. (B) Prohibitin expression and localization was monitored during yeast-to-hyphae transition in the untagged wild-type control strain and *P_TDH3_PHB2-GFP* over-expressing strain. Yeast strains were cultured in Spider medium for 3 hours. Approximately 1.0 x 10^4^ cells of each strain were selected for viewing.

**FIG S2. Slp2 localization in yeast and hyphal cells.** (A) Exponential-phase yeast cells expressing *SLP2-GFP* and *P_TDH3_SLP2-GFP* were examined with fluorescent light microscopy. Slp2-Gfp mitochondrial localization was observed in MitoTracker Red-labeled cells. (B) Fluorescent *SLP2-GFP* and *P_TDH3_SLP2-GFP* cells were quantified with flow cytometry. Untagged wild-type control cells were analyzed at identical time points and served as a negative fluorescent control. For each assay, three technical replicates were analyzed. Experiments were repeated at least three times, and data presented represents one representative experiment. *p < 0.05 and ***p < 0.001 when compared to the isogenic parental strain. (C) Co-localization tests were performed using exponential-phase *P_TDH3_SLP2-GFP* cells labeled with the following probes: 160 µM FM 4-64 (vacuole/endosome) and NucBlue Fixed Cell Ready solution (nuclear DNA). Approximately 1.0 x 10^4^ cells of each strain were selected for viewing. (D) The yeast-to-hyphae transition was observed in the wild-type untagged control strain and *P_TDH3_SLP2-GFP* over-expressing strain that were cultured in Spider medium for 3 hours.

**FIG S3. Mitochondrial and cytological analyses of stomatin mutants.** (A) ROS production was measured in stationary-phase wild-type, *slp2Δ/Δ*, and *slp2Δ/Δ/slp3Δ/Δ* mutant cells following 3 hours of 0.08% SDS treatment or dH_2_O as a control. Samples were labeled with DHR-123 and quantified by flow cytometry. (B) The following cell structures and compartments were visualized in wild-type, *slp2Δ/Δ, slp2Δ/Δ/SLP2*, *slp2Δ/Δ/slp3Δ/Δ*, and *slp3Δ/Δ* mutant cells treated with and without 0.08% SDS treatment for 3 hours: lipid droplets (LipidTOX Neutral Lipid StainTOX), cell wall (Calcofluor White), and mitochondria (MitoTracker Red; wild-type and *slp2Δ/Δ/slp3Δ/Δ* cells only).

**FIG S4. Analysis of plasma membrane permeability in stomatin mutants.** Plasma membrane permeability was analyzed in stationary-phase wild-type, *slp2Δ/Δ,* and *slp2Δ/Δ/slp3Δ/Δ* mutant cells labeled with propidium iodide (PI) following 20 hours of 0.08% SDS treatment or dH_2_O as a control. Cells were viewed with bright-field and fluorescent light microscopy (RFP).

**Supplemental Appendix.** Appendix includes yeast strain genotypes, oligonucleotide sequences, and results of phenotypic growth assays in tabular format.
